# Polyfunctional antibodies: a path towards precision vaccines for vulnerable populations

**DOI:** 10.3389/fimmu.2023.1183727

**Published:** 2023-06-27

**Authors:** Ruth A. Purcell, Robert M. Theisen, Kelly B. Arnold, Amy W. Chung, Kevin J. Selva

**Affiliations:** ^1^Department of Microbiology and Immunology, Peter Doherty Institute for Infection and Immunity, University of Melbourne, Melbourne, VIC, Australia; ^2^Department of Biomedical Engineering, University of Michigan, Ann Arbor, MI, United States

**Keywords:** antibody, allotype, polymorphism, Fc receptor, Fc function, computational modelling, vaccine design, IgG glycosylation

## Abstract

Vaccine efficacy determined within the controlled environment of a clinical trial is usually substantially greater than real-world vaccine effectiveness. Typically, this results from reduced protection of immunologically vulnerable populations, such as children, elderly individuals and people with chronic comorbidities. Consequently, these high-risk groups are frequently recommended tailored immunisation schedules to boost responses. In addition, diverse groups of healthy adults may also be variably protected by the same vaccine regimen. Current population-based vaccination strategies that consider basic clinical parameters offer a glimpse into what may be achievable if more nuanced aspects of the immune response are considered in vaccine design. To date, vaccine development has been largely empirical. However, next-generation approaches require more rational strategies. We foresee a generation of precision vaccines that consider the mechanistic basis of vaccine response variations associated with both immunogenetic and baseline health differences. Recent efforts have highlighted the importance of balanced and diverse extra-neutralising antibody functions for vaccine-induced protection. However, in immunologically vulnerable populations, significant modulation of polyfunctional antibody responses that mediate both neutralisation and effector functions has been observed. Here, we review the current understanding of key genetic and inflammatory modulators of antibody polyfunctionality that affect vaccination outcomes and consider how this knowledge may be harnessed to tailor vaccine design for improved public health.

## Introduction

Vaccines provide variable protection to different demographics as a result of complex interactions between host and environmental factors (1). This host diversity, if appropriately defined and characterised, may inform an era of precision vaccinology that accounts for inherent immunological differences between both individuals and populations (2–7). As vaccine clinical trials typically only recruit healthy adults and, unintentionally, often only from dominant ethnicities in developed countries, the data is typically not representative of vaccine efficacy in vulnerable populations (8–10). In an attempt to counter these known biases, vaccination recommendations frequently suggest prioritising early and additional doses for elderly and other immunocompromised individuals who experience reduced vaccine immunogenicity, as well as increased susceptibility to disease (11–15). Consequently, present vaccination regimens targeting specific populations are largely guided by rudimentary demographic and clinical parameters such as age and baseline health status (16–20).

However, rapid advances in molecular and systems biology along with materials science may facilitate a new frontier in population-based vaccination strategies informed by molecular mechanisms (6, 21–28). Technological and conceptual developments in vaccinology have led to numerous vaccination strategy modifications that can enhance immunogenicity and protection (1, 20, 29, 30). Concurrently, systems biology analyses of these vaccine regimens are beginning to elucidate the spectrum of protective immune interactomes (24, 27, 31, 32). These computational approaches facilitate investigation of complex biological interactions. As such, in-depth immune profiling of antibody features beyond the typically examined measures of titre and neutralisation has revealed nuanced qualitative features of antibodies that promote protection and distinguish individuals with impaired immunity (21, 33–37). Notably, a common signature associated with protection is the presence of antibody features that promote polyfunctional antibody effector functions (21, 33–37). These data may be key to informing the design of vaccines tailored to vulnerable populations.

## Importance of antibodies for vaccine-induced protection

Antibodies have been identified as a correlate of protection or control of numerous infectious diseases (38). Neutralising antibodies provide sterilising immunity by binding target epitopes leading to steric hindrance that prevents pathogen entry into host cells or inhibits toxin activity. As such, elevated neutralising titres are the principal goal of most vaccination strategies and are highly predictive of protection against many viral and bacterial diseases (38, 39). However, while neutralisation is ideal as a primary humoral defence, eliciting broadly neutralising antibodies (bnAbs) via vaccination against complex, rapidly evolving, or diverse pathogens such as malaria (40), influenza (41), human immunodeficiency virus type 1 (HIV-1) (42), and severe acute respiratory syndrome coronavirus 2 (SARS-CoV-2) (43) remains an elusive goal.

**Figure 1:**
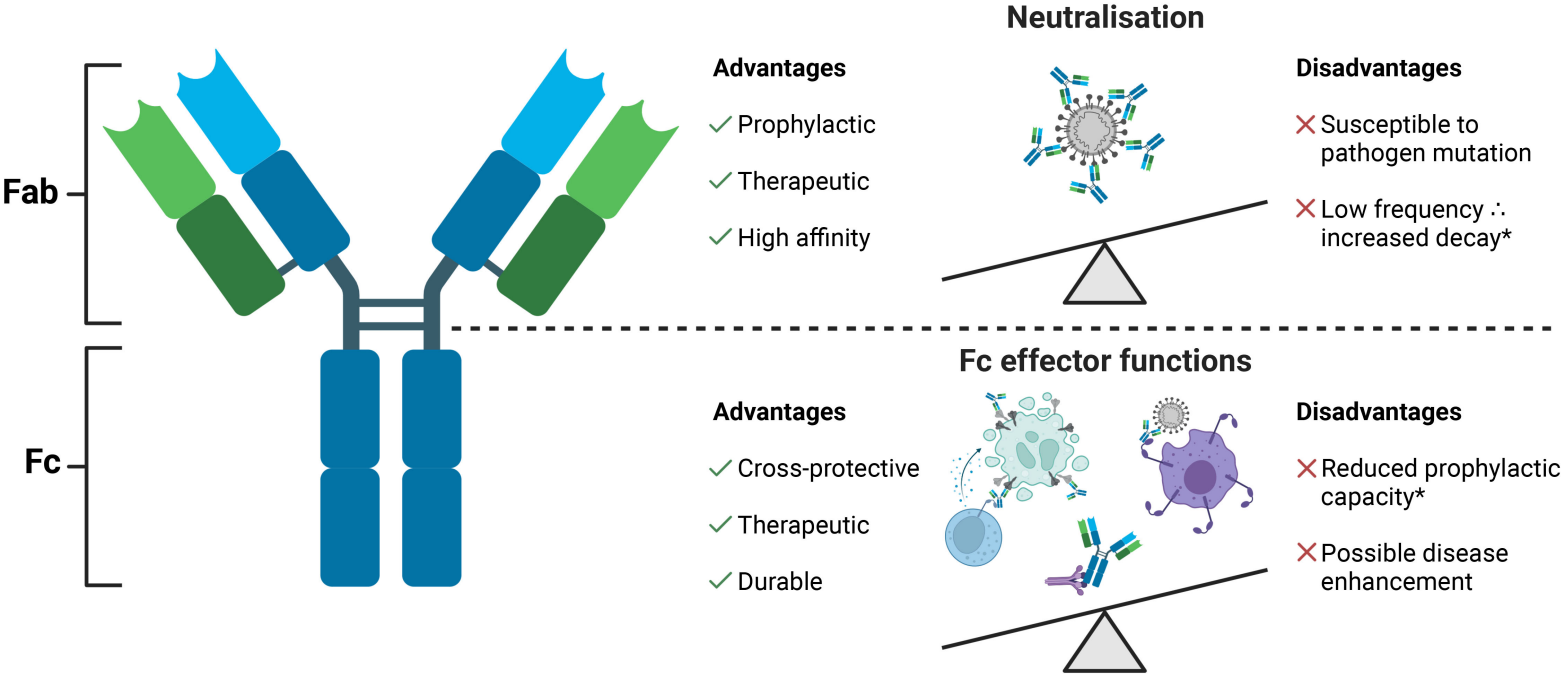
Antibodies comprise two fragment antigen binding (Fab) regions and one fragment crystallisable (Fc) region connected by a ladder-like hinge region. The Fab is responsible for antigen recognition and mediates pathogen and toxin neutralisation. The Fc engages effector cells and molecules of the innate immune system to mediate Fc effector functions. Neutralisation and Fc effector functions each have various advantages and disadvantages but largely counterbalance the shortcomings of the other. *Durable neutralisation capacity and prophylactic Fc functions observed for antibodies against some bacterial pathogens.

Antibodies comprise of two functional components: the fragment antigen binding (Fab) region which determines target specificity and is essential for neutralisation, and the fragment crystallisable (Fc) region which engages the innate immune system via numerous mechanisms (**Figure 1**). As such, Fc functions bridge the innate and adaptive immune systems by enhancing viral, bacterial, and parasite degradation and clearance as well as lysis of infected host cells in an antigen-specific manner (44). Importantly, unlike neutralising antibodies, the specificities of Fc functional antibodies are not restricted by proximity to amino acids involved in pathogen binding and fusion with host cells. Instead, Fc functional antibodies can target any conformationally accessible epitope, making these antibodies less sensitive to pathogen mutation (45–53). Nevertheless, studies of HIV-1, influenza A virus, Ebola, and SARS-CoV-2 have demonstrated that antibody specificity can substantially alter Fc potency (49, 54–57). For this reason, vaccination strategies eliciting robust extra-neutralising functions against carefully selected epitopes may be an effective approach to counter the challenges associated with bnAb generation.

## Fc effector functions enhance antibody-mediated protection

**Figure 2:**
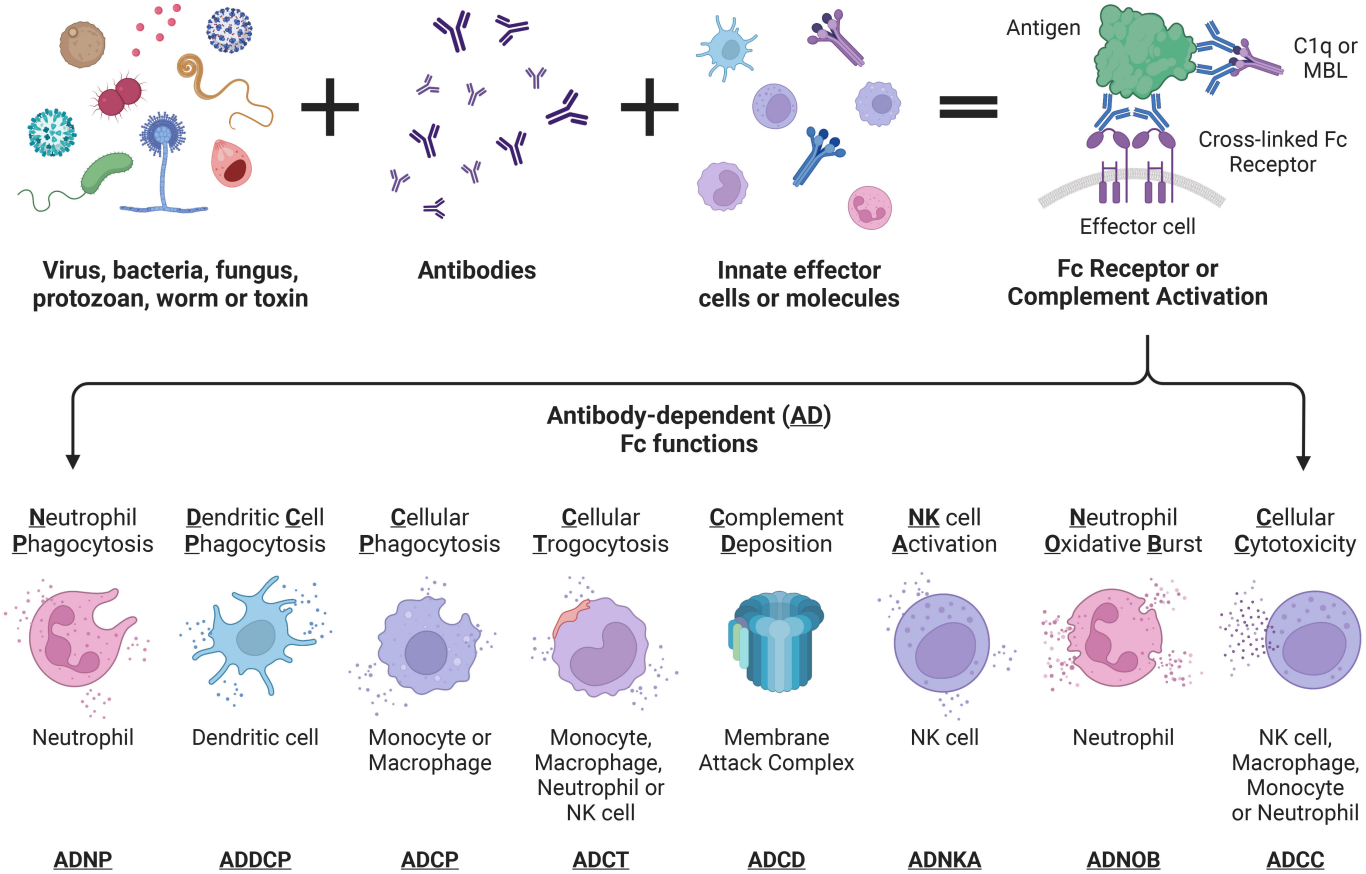
Antibody-dependent Fc effector functions referenced throughout this review. Fc effector functions are initiated upon simultaneous antibody engagement with a pathogen antigen and an innate effector molecule (complement component 1q (C1q) or mannose-binding lectin (MBL)) or Fc receptor (FcR) expressed by innate immune cells. Activation of C1q or MBL following antigen binding triggers the complement cascade leading to pathogen or infected cell death via antibody-dependent complement deposition. FcR cross-linking via antibody-antigen complexes triggers downstream signalling cascades within innate effector cells leading to pathogen killing and clearance via a range of antibody-dependent cellular effector functions, listed in the figure. Finally, these effector functions trigger downstream cytokine release which may enable further recruitment of effector cells.

Beyond neutralisation, target-bound antibodies can initiate a range of potent effector functions via simultaneous Fc region engagement with host activating Fc receptors (FcR) on various phagocytic and cytotoxic effector cells. In addition, engagement with the neonatal Fc receptor (FcRn) increases antibody half-life (58, 59). **Table 1** and **Figure 2** detail the multifaceted Fc functions that antibodies mediate along with the key effector cells and molecules involved in each process. **Figure 2** also defines abbreviations of key antibody mediated functions that are referenced subsequently throughout this review.

**Table 1:** Human Fc Receptors (FcRs) referenced throughout this review.

		Human Fc Receptors										
FcR		FcαRI	FcγRI	FcγRIIa		FcγRIIb		FcγRIIc	FcγRIIIa		FcγRIIIb	FcRn
**Cluster of Differentiation**		CD89	CD64	CD32A		CD32B		CD32C	CD16A		CD16B	–
***Gene***		*FCAR*	*FCGR1A*	*FCGR2A*		*FCGR2B*		*FCGR2C*	*FCGR3A*		*FCGR3B*	*FCGRT*
		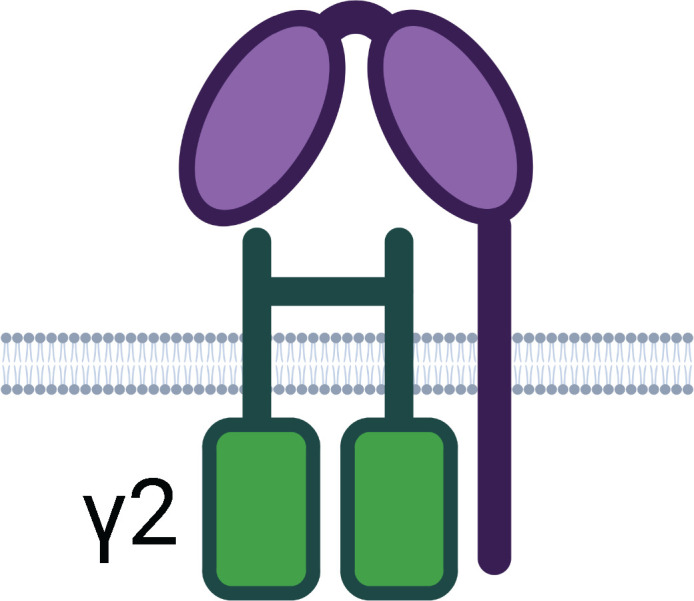	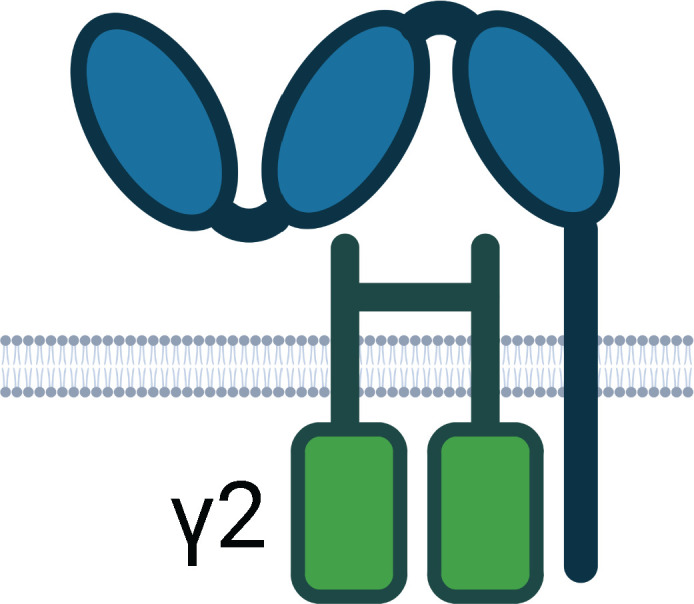	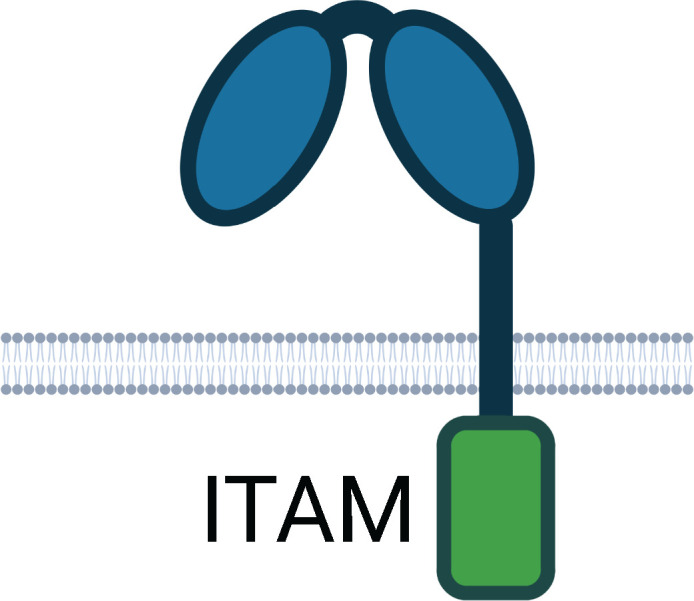		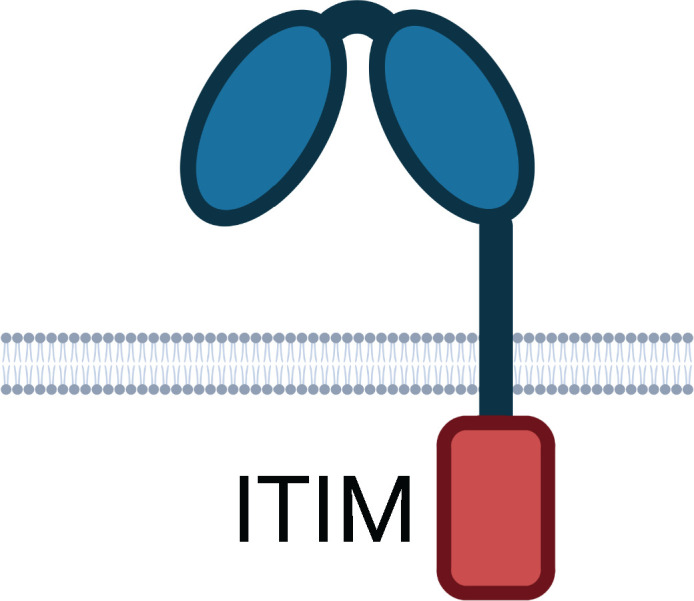		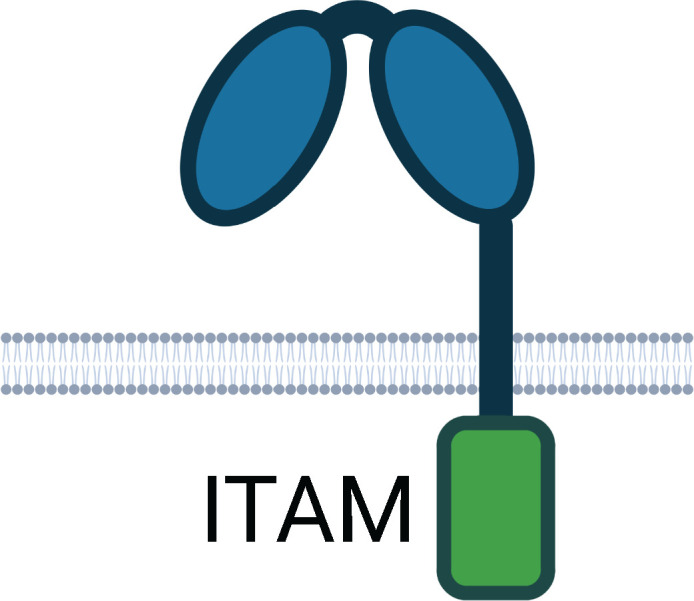	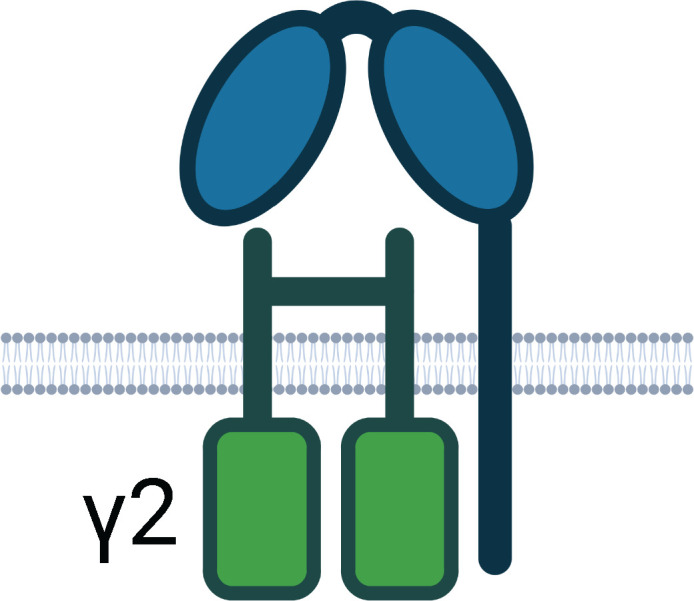		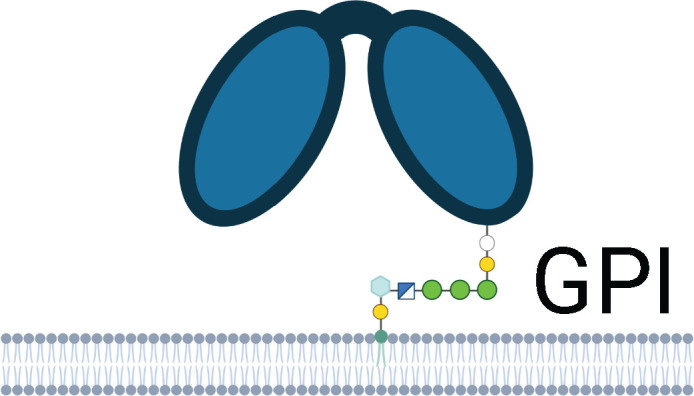	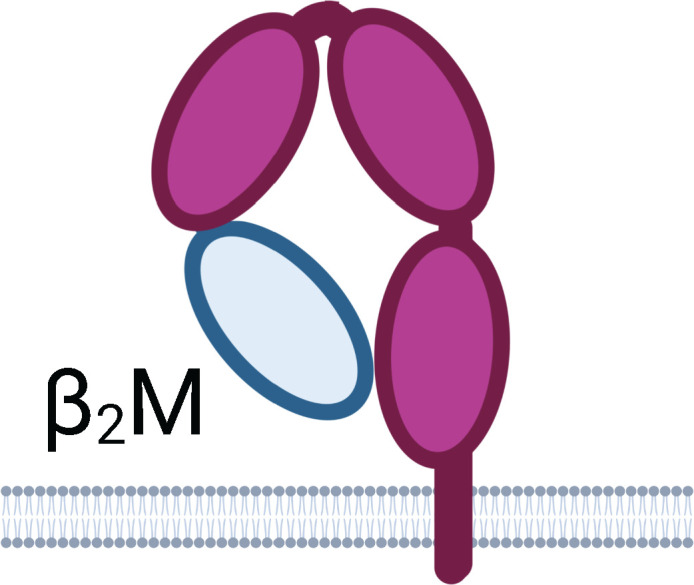
**Cellular Expression**		*Dendritic cells*, Eosinophils, *Macrophages*, Monocytes, Neutrophils	*Basophils, Dendritic cells*, *Eosinophils*, *Macrophages*, Monocytes, *Neutrophils*	Basophils, Dendritic cells, Eosinophils, Macrophages, Mast cells, Monocytes, Neutrophils, Platelets		B cells, Basophils, Dendritic cells, Eosinophils, *Macrophages*, *Monocytes*, *Neutrophils*		B cells, Macrophages, Monocytes, Neutrophils, NK cells	*Dendritic cells, Macrophages*, *Monocytes*, NK cells		*Basophils*, *Eosinophils*, Neutrophils	*B cells*, Dendritic cells, Epithelium, Endothelium, Macrophages, Monocytes, Neutrophils
**Functional Alleles**				H_131_	R_131_	I_232_	T_232_		V_158_	F_158_	NA1, NA2, SH	
**Subclass Engagement:**	**IgG1**	–	++++	+++	+++	++	ND	++	++	++	+	++++
	**IgG2**	–	–	++	++	+/-	ND	+/-	+	+/-	–	++++
	**IgG3**	–	++++	+++	+++	++	ND	++	+++	+++	++	+++
	**IgG4**	–	++++	++	++	++	ND	++	+	+	–	+++
	**IgA1/IgA2**	+++	–	–	–	–	–	–	–	–	–	–
**Key Functions**		Activation & ITAMi inhibition	Activation	Activation & ITAMi inhibition		Inhibition		Activation	Activation & ITAMi inhibition		Decoy & Activation	IgG recycling & Transcytosis

Schematics represent FcR immunoglobulin-like domains and, in the case of FcRn, β_2_M (beta 2 microglobulin), as oval structures, along with associated signalling subunits or membrane anchoring domains. γ2: gamma chain; ITAM: immunoreceptor tyrosine-based activation motif; ITIM: immunoreceptor tyrosine-based inhibitory motif; GPI: glycosylphosphatidylinositol anchor. Cellular Expression lists cell types in which the receptor has been identified; italicised cell types indicate that FcR expression may be either low, inducible, or only present on a subset of the indicated population. NA1: human neutrophil-specific antigen 1a (HNA-1a); NA2: human neutrophil-specific antigen 1b (HNA-1b); SH: human neutrophil-specific antigen 1c (HNA-1c). Subclass engagement ranks IgG subclass affinities for each respective FcR; +/- indicates very low to absent binding. Data compiled from (44, 58, 60–66).

Fc effector functions are appreciated as correlates of protection for multiple bacterial pathogens (38). Bactericidal antibodies underpin protection following meningococcal vaccination (67), and vaccine-induced antibody-dependent neutrophil phagocytosis (ADNP) is recognised as a correlate of protection against *Streptococcus pneumoniae* (68). Fc effector functions have also been highlighted as a key correlate of malaria protection in studies of the RTS,S/AS01 vaccine (69–72). Antibody titres alone were not associated with protection, however, protection following parasite challenge was predicted by capacity for antibody-dependent cellular phagocytosis (ADCP) and FcγRIIIa engagement (69, 71) as well as an immunoglobulin G (IgG) subclass distribution skewed towards IgG3 and away from IgG2 which would favour enhanced FcR engagement and effector functions (70, 72). Furthermore, in the case of HIV-1 (73), human papillomavirus (HPV) (74, 75), influenza (76, 77), and SARS-CoV-2 (78), neutralising antibodies do not fully explain vaccination-induced humoral protection, suggesting a pertinent role for Fc effector functions in antibody-mediated immunity (51, 79, 80). This phenomenon has been well-described for the only moderately protective HIV-1 vaccine trial, RV144, which demonstrated partial efficacy in the absence of bnAbs (73, 81); further antibody profiling indicated this phenomenon to be a consequence of robust Fc effector functions (73, 82, 83). Similarly, protection from respiratory syncytial virus (RSV) is poorly predicted by serum IgG levels or neutralising titres. Instead, Fc effector functions may be a better correlate of vaccine-induced protection (84, 85).

The importance of Fc functions in protection against pathogens has been demonstrated in animal models of HIV-1 (86), SARS-CoV-2 (87–89) and influenza challenge (49) in which neutralising monoclonal antibodies (mAbs) required Fc-functional capacity for optimal prophylaxis and treatment. The value of Fc functions was demonstrated in macaque models of HIV-1 infection whereby administration of neutralising mAbs with an Fc LALA mutation (two consecutive leucine to alanine substitutions which abolish antibody binding to FcγRs) impaired protection compared to intact mAbs (86). In the case of SARS-CoV-2, humanised mice and Syrian hamsters administered Fc-functional mAbs exhibited reduced viral load and immunopathology compared to those administered mAbs with an Fc LALA mutation (88). These protective effects were only observed in the presence of monocytes, but the absence of neutrophils or NK cells had no effect on weight loss, indicating a dominant role for ADCP (88). In addition, mAbs containing the GASDALIE mutation that promotes enhanced FcγRIIIa binding showed improved protection against lethal SARS-CoV-2 challenge compared to wild-type mAbs (87). In the case of influenza, while bnAbs against the variable head region of the hemagglutinin (HA) protein did not require Fc functional capacity for protection, bnAbs directed against the conserved stalk region required FcγR-driven antibody-dependent cellular cytotoxicity (ADCC) to confer protection against lethal H1N1 challenge (49). Given the importance of cross-reactive anti-HA stalk antibodies to counter the high mutation rates of influenza, Fc functions have great value in influenza protection (90).

Immune responses associated with reduced infection risk and severity can guide vaccine development. Indeed, the parallel identification of ADCP and antibody-dependent natural killer cell activation (ADNKA) as correlates of protection against malaria in both vaccinated and unvaccinated individuals (69, 71, 91–93) suggests a level of homology between the protective mechanisms induced by vaccination and those required for disease resolution. Similarly, ADNKA has been associated with protection against RSV following vaccination or infection (84, 94, 95). In addition, enhanced ADCC is associated with HIV-1 viremic control (96–99) and was also identified as a correlate of protection following RV144 HIV-1 vaccination (34, 82). Given the wide-ranging benefits of a coordinated Fc response, it follows that robust Fc functions are implicated in protection against most diseases for which vaccines are licenced or in clinical trial (**Table 2**). Furthermore, for highly fatal infections, such as Ebola virus disease (57, 193) and Marburg virus disease (159), Fc effector functions promote protection and survival, as well as reduction of long-term sequelae. Therefore, targeting generation of broad and highly potent Fc effector functions is likely a valuable goal of many vaccines currently under development.

**Table 2:** Infectious diseases for which Fc effector functions are involved in protection or antimicrobial activity, and for which vaccines are licenced or in clinical trial.

Pathogen (Infectious disease)	Evidence for Fc Effector Function Involvement		
	Fc Effector Functions	Ab Features & FcR Interaction	Outcome (Experimental model)
***Bordetella pertussis*** *(Pertussis/Whooping cough)*	ADNP & ADNOB	Opsonic IgG & IgA; FcγRIIa, FcγRIIIb & FcαRI	**Anti-bacterial activity** (human patient cohort) (100)
	ADCD	Bactericidal IgG3	**Anti-bacterial activity** (human patient cohort) (101, 102)
	ADCD, ADNP & ADNOB	Opsonic IgA, FcαRI & FcγR	**Protection & bacterial clearance** (mouse model) (103–107)
**Dengue virus** (Dengue fever)	ADCD, ADCP & ADNKA	↑ IgG4; Coordinated FcγRIIIa engagement	**Protection** against symptomatic infection (human patient cohort) (108)
	↑ NK cell ADCC, ADNKA	Coordinated FcγRIIIa engagement	↓ **Symptomatic** & **Secondary DENV-3**, but not DENV-2, **viremia** (human patient cohort) (108, 109)
	↑ ADCC	↑ IgG1 afucosylation; ↑ FcγRIIIa binding	↑ **Disease severity, ADE activity** (human patient cohort) (110–112)
***Haemophilus influenzae*** **serotype b (**Hib)	ADCD, ADNP	Bactericidal and opsonic IgG1 & IgG2	**Bacterial lysis** (human patient cohort) (113–115)
	ADCD, ADNP	Bactericidal IgM and IgG & opsonic IgG	**Protection** (rat model) (116)
	ADCD & ADCP or ADNP		**Bacterial clearance** (mouse model) (117)
**Hepatitis B virus** (Hepatitis B)	NK cell ADCC		**Resolution** & **remission** (human patient cohort) (118, 119)
	↓ ADCD	↓ FcγRIIb	**Chronic disease** & ↑ ALT, AST (markers of liver damage) (human patient cohort) (120, 121)
		↓ IgG galactosylation	**Chronic disease** & **cirrhosis** (human patient cohort) (122)
	NK cell and macrophage ADCC & macrophage ADCP		**Protection** (*in vitro* and mouse model) (123)
**Herpes Simplex Viruses 1 & 2**	↑ ADCC		↓ **Neonatal disease severity** (human patient cohort) (124)
	↑ ADCC	↑ IgG1 & ↓ IgG3	**Antiviral activity** during **chronic infection** (human patient cohort) (125)
	ADCC	Non-neutralising antibodies	**Protection** following vaccination (mouse model) (126, 127)
		F(ab)’2 fragments	↓ **Protection** compared to intact IgG (mouse model) (128, 129)
**Human cytomegalovirus**	ADCP	Non-neutralising IgG1 & IgG3	**Vaccine immunogenicity** (human vaccine trial) (130)
	NK cell expansion	FcγRIIIa expression	**Infection control** (human case study) (131)
	ADCP & NK cell ADCC		**Antiviral activity** (in vitro) (132, 133)
**Human immunodeficiency virus 1** (Acquired immunodeficiency syndrome)	↑ ADCC, ADCP, ADNKA & ADCD	↑ IgG3, coordinated IgG1 & IgG3, ↓ IgG2 & IgG4, ↓ IgA	**Protection** (human vaccine trials) (34, 73, 82, 83, 134, 135)
	↑ ADCVI*		↑ **Protection** (human vaccine trial) (136)
		↑ Fucosylated, agalactosylated IgG; ↑ FcγRIIIa engagement	**Disease control** compared to acute infection (human patient cohort) (79)
	↑ ADCC		↓ **Progression** (human patient cohort) (99, 137, 138)
	↓ ADCC	↓ FcγR engagement by neutralising mAbs	↓ **Protection** (macaque model) (86)
	↑ ADCC, ADCD, ADCP, ADNKA & ↑ polyfunctionality		↑ **Protection** (macaque model) (139–143)
**Human papillomavirus** (HPV)	ADCD	IgG3	**Vaccine immunogenicity** (human clinical trial) (75)
	ADCP or ADNP; neutrophils	FcγR	**Protection** (mouse model) (144, 145)
**Influenza A virus & Influenza B virus** (Influenza)	ADCC		**Survival of severe disease** (human patient cohort) (146)
	ADCC, ADNKA & ADCP	Non-neutralising mAbs; FcγRIIIa & FcγRIIa	**Cross-reactive antiviral activity** (human patient cohort) (51, 52, 147–153)
	ADCC	FcγRIIIa	**Protection** (mouse model) (49, 154)
	ADCC & ADCP	Non-neutralising antibodies	**Protection** (mouse model) (155)
	ADCP	FcγRI & FcγRIII	↑ **Protection, viral clearance** and ↓ **disease susceptibility** (mouse model) (156, 157)
**Lassa virus** (Lassa fever)	ADCC & ADCP	FcγRII & FcγRIII	**Protection** following vaccination (mouse and guinea pig model) (158)
**Marburg Virus** (Marburg Virus Disease)	ADCP, ADNP & ADNKA	IgG3	**Protection against death** (159)
**Measles morbillivirus** (Measles)	ADNP, ADCP, ADCD, & ADCC		**Vaccine immunogenicity** (human patient cohort) (160)
***Mycobacterium tuberculosis*** (Tuberculosis)	↑ ADNKA & ADCC	↑ IgG afucosylation	**Latent infection** (human patient cohort) (53)
	↑ ADCP		**Active infection** (human patient cohort) (53)
	↑ ADNKA	↓ IgG sialylation	↓ **Disease susceptibility** (human patient cohort) (161)
		↑ IgG3	↓ **Recurrent infection** (human patient cohort) (162)
		↓ Inhibitory FcγRIIb expression	↓ **Bacterial burden** & ↑ **survival** (mouse model) (163)
***Neisseria meningitidis*** (Meningococcal disease)	ADCD, ADNP & ADNOB	Bactericidal and opsonic IgG1 & IgG3	**Vaccine immunogenicity** (human clinical trial) (164–166)
		↑ Bactericidal antibodies	↓ **Disease susceptibility** (human patient cohort) (167)
	ADNOB, ADCD & ADNP	Bactericidal IgG1, IgG3 & IgA	**Infected cell lysis** (*in vitro*) (168)
***Plasmodium falciparum*** (Malaria)	ADNP & ADCD		**Protection** following vaccination (human challenge trial) (71)
	ADCP & ADNKA	FcγRIIIa engagement	**Protection** following vaccination (human challenge trial) (69, 92)
	ADCP		**Protection** following previous exposure (human challenge trial) (91)
	ADNKA	IgG1 & IgG3	**Protection** (human challenge trial) & ↓ **clinical episodes** (human patient cohort) (93)
	ADCP & ADNOB	IgG1 & IgG3	**Protection** in endemic regions (human patient cohort) (169–171)
	ADCD	IgG3	**Vaccine immunogenicity** (vaccine clinical trial) & ↓ **clinical episodes** (human patient cohort) (172)
***Salmonella enterica* serotype Typhi** (Typhoid fever)	ADCC	IgA	**Protection** (human patient cohort) (173)
	ADNP & ADNOB	FcαRI	**Vaccine-induced protection** (human patient cohort) (174)
	ADCC	FcgRI, II & III	**Vaccine-induced protection** (mouse model) (175, 176)
**SARS-CoV-2** (COVID-19)	↑ADCP & ADNKA	Coordinated FcγR engagement	↑ **Survival of severe disease** (human patient cohort) (177)
	ADCC & ADCP		**Cross-reactive antiviral activity** (human patient cohort) (47, 48)
	ADCP, ADCT, ADCC & ADNKA	FcγRIIa & FcγRIIIa engagement	↑ **Antiviral durability** compared to neutralisation (human patient cohort) (178)
	ADCC & ADCP; macrophages	↑ FcγRIII engagement	↓ **Mortality** & **pathology** (mouse and hamster models) (56, 87–89)
***Streptococcus pneumoniae*** (pneumococcal disease)	ADNP	IgG1, IgG2 & serum IgA; FcγRIIa & FcαRI	**Anti-bacterial activity** (human patient cohort) (179, 180)
	ADNP or ADCP	↑ Opsonic IgG	↑ **Protection** (Mouse model) (181)
**Respiratory Syncytial Virus** (RSV)	ADCD, ADNKA, ADCP & ADNP		**Vaccine immunogenicity** (human challenge trial) (84)
	ADCP & ADNP	↑ FcγRIIb, likely as a surrogate for ↑ FcγRIIa & FcγRIIIa engagement	**Protection** (human challenge trial) (84)
	↓ Global Fc functions	↑ IgG4; ↑ IgG digalactosylation & fucosylation	↓ **Protection** (human challenge trial) (84)
	↓ ADNKA	↑ IgG fucosylation	**Severe infection** (human patient cohort) (94)
**Varicella-zoster virus** (Chickenpox or Shingles)	ADCC		**Early-stage viral control** (human patient cohort) (182)
	NK cell & monocyte ADCC		**Clearance of infected cells** (*in vitro* and human patient cohort) (183, 184)
***Vibrio cholerae*** (Cholera)	ADCD	Vibriocidal antibodies	**Long-term protection** (human clinical trial & human patient cohort) (185, 186)
**Yellow Fever virus** (Yellow Fever)	ADCC	FcγRIIIa	**Cross-reactive protection** following Japanese encephalitis vaccination (mouse model) (187)
	NK cell ADCC		**Protection** (mouse model) (188)
		F(ab’)2 fragments	↓ **Protection** compared to intact IgG (mouse model) (188)
***Zaire ebolavirus*** (Ebola virus disease)	ADCD & moderate ADNKA		**Survival** (mouse model and human patient cohort) (189)
	ADCD, ADNP &ADCP	IgG1	**Survival** (nonhuman primate model) (190)
	ADCD & ADCC		↑ **Protection** (macaque model) (191)
	NK cell ADCC		↑ **Protection** (*in vitro* & mouse model) (192)
	ADCP, ADNP & ↑ polyfunctionality		↑ **Protection** (*in vitro* & mouse model) (57)

*Antibody-dependent cell-mediated virus inhibition (ADCVI) is a measure of FcγR-mediated antiviral activity that accounts for antibody polyfunctionality

## Advantages of Fc mediated functions

Even when sterilising immunity is achievable via vaccination, neutralisation escape is frequent as a result of viral evolution. The effect of even a few amino acid mutations upon neutralisation has been extensively studied in the face of SARS-CoV-2 variants, where significantly weaker neutralising titres are observed against Omicron subvariants in comparison to the ancestral strain, and this remains true despite repeated vaccine boosts (194–196). Although boosting with Omicron BA.5 or BA.4/BA.5 adapted bivalent booster vaccination recovers neutralisation of the BA.4/BA.5 variant, neutralisation capacity is again lost against more recently emerged variants such as BQ.1.1 and XBB.1 (195). Given that perpetually updating vaccines to protect against continuously emerging viral variants is highly challenging, design of vaccines eliciting broadly protective functions, such as Fc-effector functions, is warranted. Indeed, the extent of this Fc functional antibody cross-reactivity is demonstrated by the ability of a chimeric Japanese encephalitis virus (JEV) vaccine (consisting of JEV structural genes upon the yellow fever virus vaccine YFV-17D scaffold) to protect mice against lethal yellow fever virus challenge via FcγRIIIa-mediated ADCC (187). Of note, although ADCC-mediating antibodies may exert selective pressure on HIV-1 evolution (197), the likelihood of Fc functions to drive evolution of viral escape mutations is reduced compared to that of neutralising antibodies (198, 199). This constraint of neutralisation escape mechanisms further supports prioritisation of Fc functions in vaccine development.

Fc-functional antibodies are also more durable than neutralising antibodies (178) given the increased abundance of non-neutralising antibodies, which, for example, may constitute up to 95% of antibodies against the SARS-CoV-2 spike protein (46). In human cohorts, a study characterising various antibody features of convalescent plasma from 36 mild-moderate coronavirus disease 2019 (COVID-19) recovered patients up to five months post-infection, 100% and 94% of participants maintained ADCP and ADCC functions, respectively, while neutralisation was only detectable in 70% of participants (178). Independent studies have also detected persistence of neutralising antibodies against SARS-CoV-2 five months following infection (200), however, the longevity of the response is dependent upon disease severity (201). Similar to the kinetics of post-infection responses, neutralising antibodies induced by SARS-CoV-2 vaccination have been found to decay within four to six months, particularly against SARS-CoV-2 variants of concern (202) and among immunocompromised populations (43). As such, this data reinforces the value of Fc functions in protecting vulnerable populations against evolving pandemics.

A further benefit of Fc functional antibodies is their dual capacity for both protection against infection as well as control of disease through collaboration with neutralising antibodies and T cells, respectively, as demonstrated by both mechanistic (203) and systems serology (78, 204) studies of SARS-CoV-2. Furthermore, enhanced Fc engagement with FcγRIIa supports increased dendritic cell maturation and CD8^+^ T cell responses, facilitating improved protection against influenza (205). In the case of SARS-CoV-2, although neutralising titres remain predictive of protection against symptomatic disease in the face of variants (206), with up to log-fold reductions in neutralisation (195), cross-reactive Fc functions likely mitigate, at least in part, the severe disease outcomes that might be expected with such drastic losses in neutralisation. As such, it is likely that, along with T cell responses (207), highly conserved Fc effector functions directed against novel variants (47, 48) may protect against severe outcomes.

## Fc modifications predict effector functions

Despite the Fc portion belonging to the antibody constant region, numerous Fc modifications contribute to antibody diversity (208, 209). Antibody quality can be enhanced by heritable and inducible genetic variation of the Fc region in the form of antibody isotypes, subclasses, and allotypes, as well as post-translational Fc modifications such as glycosylation. This variation can greatly impact FcR interactions, and therefore, alter potency of Fc functions, with antibody isotype and subclass modulation typically having the greatest effects (208). Importantly, changes to the abundance of various antibody isotypes, subclasses, and glycosylation patterns can be induced via both vaccination and disease (110, 210–213). Critically, regulation of these Fc modifications is a complex, multilayered process influenced by a range of innate and adaptive immune cells and cytokines (214–218).

### Isotypes and subclasses

**Figure 3:**
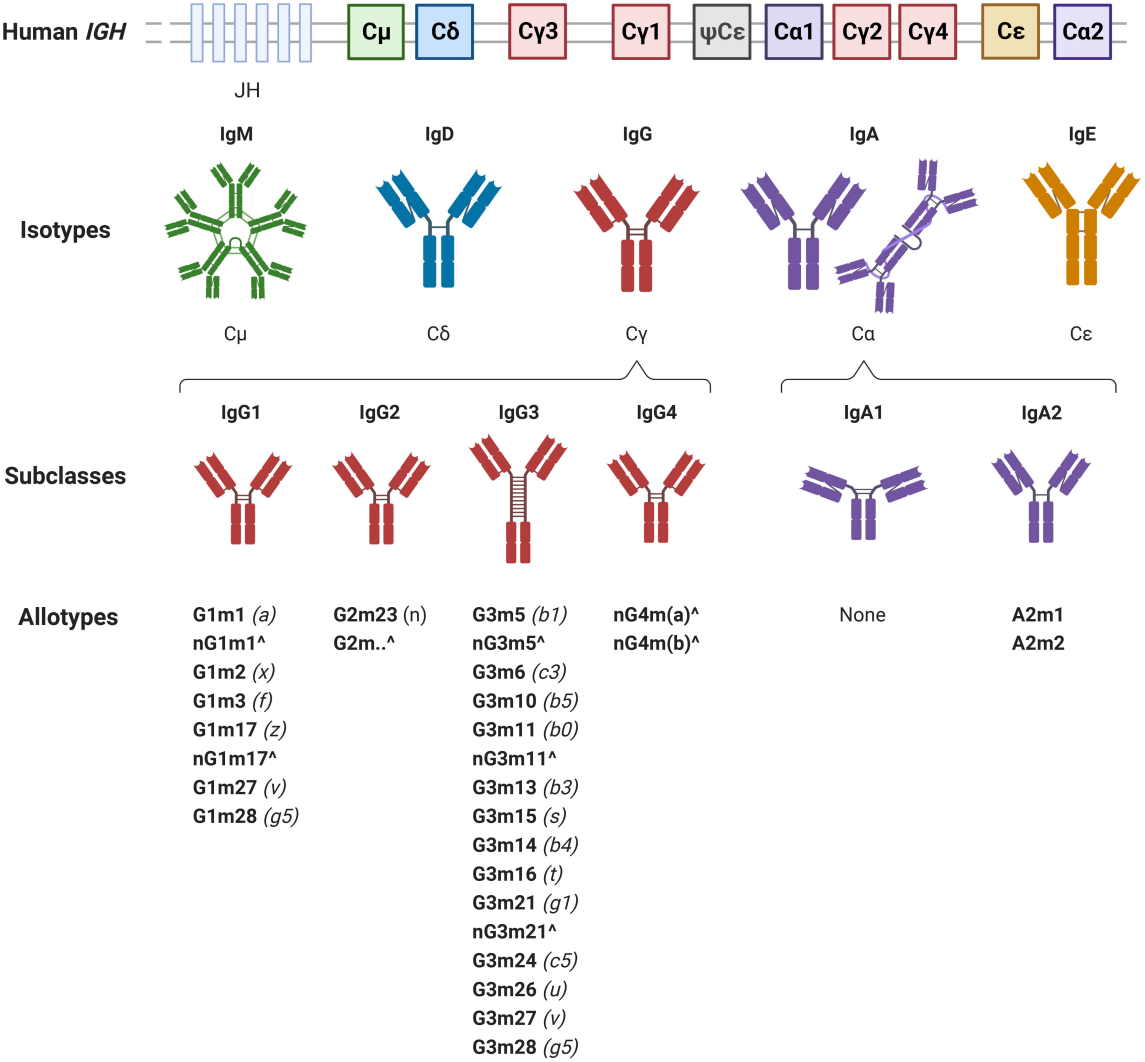
The immunoglobulin heavy (*IGH*) locus encodes the constant regions of immunoglobulin (Ig) M, IgD, IgG, IgA, and IgE. The constant heavy genes are located downstream of the joining region heavy (JH) genes. One pseudogene (ψCϵ) is also located within the *IGH* locus. IgG and IgA comprise four and two subclasses, respectively. Additional antibody variation is introduced by the single nucleotide polymorphisms which, alone or in combination, define a range of IgG1, IgG2, IgG3, IgG4, and IgA2 allotypes. Allotypes are listed according to the WHO/IUIS nomenclature in bold, followed by the previous alphabetical notation italicised in brackets. ‘Gm’ or ‘Am’ designates a marker of IgG1-4 or IgA, respectively, followed by a number corresponding to the named allele. **^**The prefix ‘n’ or suffix '..' indicates the absence of the allotypic marker at the named allele; these are referred to as isoallotypes and contain an amino acid distinct to the subclass but common across the isotype. (Note that ‘nG1m1’ may be written as ‘G1m-1’ to indicate the absence of the G1m1 allotype). Each named allele is located at a distinct amino position except G1m17 and G1m3 which represent allotypes at the same position.

Upon B cell activation, early IgD^+^ and IgM^+^ lymphocytes undergo affinity maturation and DNA recombination in the form of class switch recombination (CSR). CSR enables selective usage of a single immunoglobulin heavy constant (*IGH*) gene (Cμ through to Cα2) per transcript, with a bias towards downstream genes with increasing antigen exposure. As such, this process converts antibody constant regions to more mature isotypes (**Figure 3**), generating higher affinity, Fc-functional IgA, IgG, or IgE antibodies, depending on the antigen encountered. IgA production is largely driven by mucosal antigen exposure, with IgA1:IgA2 subclass ratios partially dependent on the anatomical site of exposure (219) and host age (220). IgG1-4 subclasses may be selectively induced depending on antigen characteristics, exposure frequency, or host age (213, 220, 221). CSR is further influenced by signalling molecules as well as numerous immune cell subsets, including antigen presenting cells, conventional T cells, and unconventional T cells, as discussed in detail in the following reviews (214–218). Importantly, cytokines secreted by CD4^+^ helper T (T_h_) cells, including interleukin (IL)-4, IL-10, IL-13, and IL-21, have dominant roles in class switching to IgG, with IgG subclass distributions influenced by T_h_ cell subset ratios and innate immune cells (222–227).

IgG3, followed by IgG1, has the highest affinity for FcγRs and, consequently, the greatest Fc-functional capacity, granting this subclass its so-called ‘cytophilic’ nature (228, 229) (**Table 1**). As such, elevated levels of IgG1 and IgG3 are correlated with superior protection against a range of diseases following infection or vaccination (229, 230). The robust polyfunctionality of IgG3 can be further complemented by the increased neutralisation potency observed for certain IgG3 variants (231–233). On the other hand, IgG2- or IgG4-skewed responses with reduced Fc functionality have been associated with non-protective HIV-1 trials (34, 82, 234). However, in diseases such dengue fever in which a hyperinflammatory response can be pathological, increased induction of IgG4 is more protective (108).

CSR is coordinated by multiple enzymes with dual functionality in somatic hypermutation (SHM)—the process enabling antibody Fab region diversification. Most notably, activation-induced cytidine deaminase (AID) initiates CSR and SHM and is indispensable for these mechanisms (235). The importance of AID to polyfunctional antibody responses is demonstrated by the positive association of AID expression with increased neutralisation breadth, IgG subclass diversity, and Fc responses following HIV-1 infection (236), as well as the diminished production of mature isotypes and reduced affinity maturation in individuals with impaired AID expression, such as the elderly or those with chronic inflammatory conditions (237–239).

### Allotypes

Evolutionary pressures imposed by pathogens, particularly malaria, upon human populations for millennia have made immunoglobulin genes are a key target for genetic diversification mechanisms (240–242). As such, single nucleotide polymorphisms (SNPs), and combinations thereof, within the antibody constant region introduce a further layer of variability to the variable Fc-functional capacity of IgG subclasses. Initially defined via serological detection methods and termed ‘allotypes’, these antibody variants now form part of a continuously growing collection of *IGH* gamma (*IGHG*) chain alleles (243–245). IgG1 possess four classical allotypic markers present only in the IgG1 subclass, as well as two supernumerary markers occurring in IgG3 in some populations; one allotype is present in IgG2, and 13 IgG3 allotypes exist, including the two IgG1 supernumerary markers. In addition, two IgG4 isoallotypes which possess amino acids unique within the subclass but occurring in other antibodies across the isotype have been identified (245, 246) (**Figure 3**). Notably, *IGHG* genes are inherited in a Mendelian fashion and are in linkage disequilibrium such that specific allotypes are typically inherited within haplotype blocks (247–249). This is particularly evident in IgG3 which exhibits exceptional allelic diversity, and as such, IgG3 nomenclature is simplified to indicate commonly inherited combinations of alleles, annotated as G3m5* or G3m21*, for example (229). Notably, the G1m1 allotype is commonly inherited with G1m17 and, to a slightly lesser extent, G3m21* (250). As such the antithetical high prevalence allotype is Gm-1,3,5*.

Notably, advances in molecular biology and inclusion of Indigenous populations in biomedical research has enabled recent identification of additional polymorphisms (209, 243, 251). This extensive antibody diversity likely reflects the variable evolutionary selective pressures of different disease burdens imposed upon distinct populations, resulting in the selection of numerous low frequency polymorphisms in genetically isolated populations (252). However, a subset of dominant allotypes underpin variable responses to infection and vaccination. Across a diverse array of viral, bacterial, and protozoan infections, these IgG variants are associated with altered disease susceptibility possibly driven by IgG subclass distribution and titres of antigen-specific antibodies (**Supplementary Table**). In addition, IgG allotypes are reported to influence subclass titres and distribution of total IgG (253, 254). These variations to subclass distribution are suggested to impact Fc effector functions if antibody subclasses with reduced Fc functional capacity, such as IgG2 and IgG4, are expressed at the expense of more functional subclasses such as IgG3.

Allotype-associated regulation of Fc-functional capacity remains under-studied (244). However, recent monoclonal antibody studies revealed that IgG3 allotypes bind FcγRIIIa with different affinities and, therefore, have varied capacity to trigger ADCC, ADCP, and antibody-dependent cellular trogocytosis (ADCT) (233, 255). In addition, substitution of arginine to histidine at position 435 in some IgG3 allotypes can triple the half-life of this typically short-lived subclass via enhanced binding to FcRn (256–258). This polymorphism has been associated with increased transplacental transfer of malaria-specific IgG and improved protection against malaria during infancy (257). However, the mechanisms by which other IgG polymorphisms confer altered protection against infectious diseases or why allotypes are associated with drastic changes in IgG subclass expression remains poorly understood and warrants further investigation.

### N-linked glycosylation

**Figure 4:**
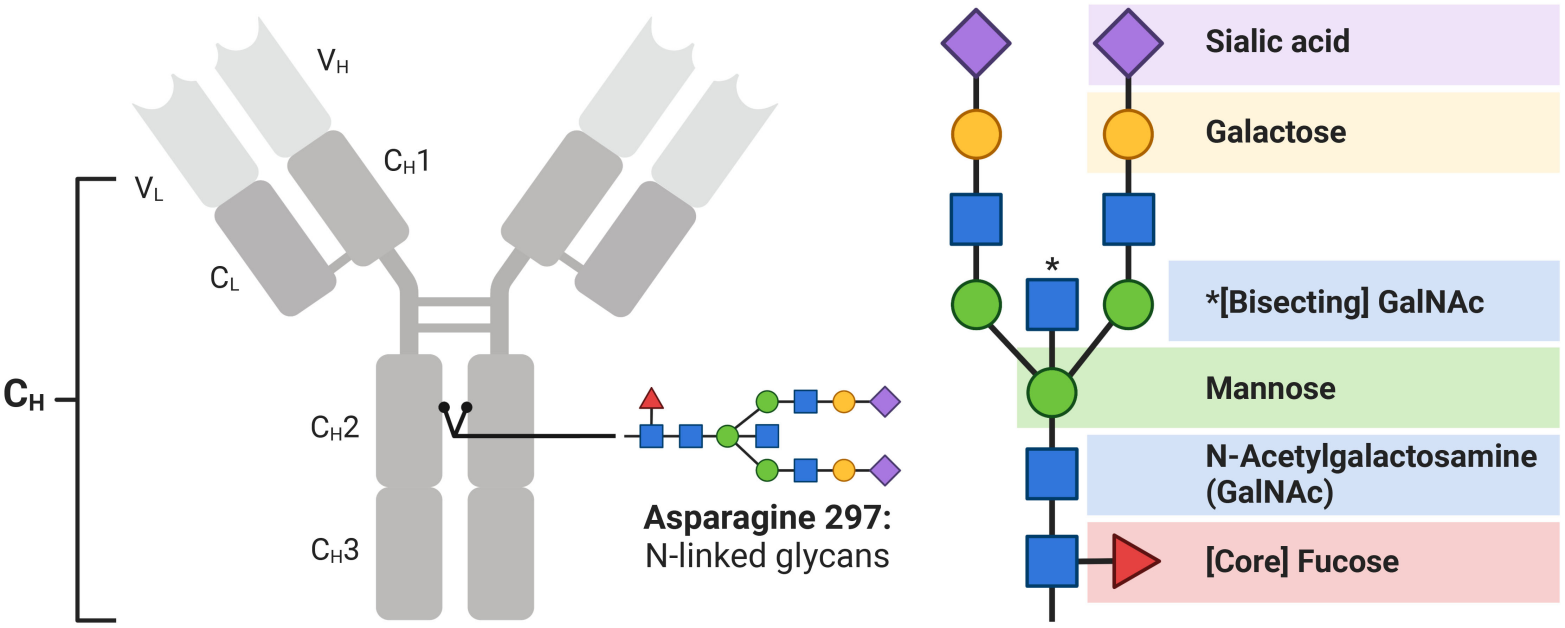
IgG is post-translationally glycosylated. Biantennary N-linked glycan chains are added at asparagine 297 within the Fc portion of the constant heavy (C_H_) regions of IgG. Two N-acetylglucosamine (GlcNAc) subunits and three mannose subunits form two branching structures upon which additional GlcNAc, followed by galactose and then sialic acid are added. Fucose can be linked to the N_297_ proximal GlcNAc and is present on the majority of human IgG.

Beyond genetic polymorphisms and gene rearrangements which impact protein sequence and structure, post-translational glycosylation of IgG is an additional key regulator of Fc functions. Enzymatic addition of polysaccharide chains to the antibody Fab, hinge, and Fc regions can modify both antigen specificity and Fc receptor engagement, with Fc glycosylation at asparagine 297 (N_297_) within the constant heavy chain two (C_H_2) region influencing antibody polyfunctionality most substantially via modulation of Fc effector functions (259–263). Typical IgG Fc glycan chains are biantennary in nature, consisting of N-acetylglucosamine (GlcNAc) subunits from which mannose subunits form two branching structures allowing for the orderly addition of further GlcNAc, followed by galactose and then sialic acid. In addition, monomeric fucose can be linked to the N_297_ proximal GlcNAc (**Figure 4**). Variations to this N-linked glycosylation are associated with modulation of the inflammatory capacity of IgG, given the associated changes in affinity during IgG-FcγR interactions (264). It follows, therefore, that Fc glycosylation patterns predict antibody effector functions (265).

Fucose has the best characterised role in modulating IgG-FcγR interactions and downstream Fc effector functions. A unique carbohydrate-carbohydrate interface exists between the glycans of afuscosylated IgG and FcγRIIIa that greatly enhances affinity compared to when core fucose is present and consequently interferes with formation of this interface (266). As such, afuscosylation is associated with upregulated FcγRIIIa signalling and enhanced ADCC and possibly ADCP (266–271).

Galactose is reported to modulate Fc effector functions, with increased galactosylation associated with increased IgG1 and IgG3 binding to complement component 1q (C1q) and, therefore, enhanced antibody-dependent complement deposition (ADCD) (271, 272). Increased galactosylation is also correlated with enhanced FcγRIIIa engagement and ADCC (271, 273, 274). However, galactose only subtly improves affinity for FcγRIIIa and does not further promote ADCC in an environment of highly afucosylated IgG (273, 275). Most critically, as galactose is the building block required for addition of sialic acid, it is essential for the anti-inflammatory properties associated with sialyation (276).

Sialic acid may inhibit FcγRIIIa binding and activation by IgG, thereby downregulating ADCC (263). However, the mechanism by which this occurs remains disputed owing to conflicting structural data (277–279). Alternatively, sialic acid may dampen inflammation by upregulating expression of inhibitory FcγRIIb (280, 281) or shifting IgG Fc receptor specificity towards C-type lectins that mediate anti-inflammatory functions (277, 282, 283). Nevertheless, these explanations which purportedly underpin the anti-inflammatory properties of intravenous immunoglobulin (280–283), are also contested (284, 285). Importantly, given the dominant role of afucosylation in modulating ADCC via enhanced FcγRIIIa binding, Fc sialyation has been suggested to only adversely impact the ADCC capacity of fucosylated, but not afucosylated IgG (286).

Critically, Fc glycosylation is under the control of a combination of genetic, hormonal, and cytokine regulatory mechanisms (287) which remain to be fully elucidated. However, IL-6 and IL-23 play relatively well-described roles in modulating Fc sialyation in mice (288, 289). IL-6 and IL-23 promote IL-17 secretion by T follicular helper 17 (T_fh_17) cells which downregulates β-galactoside α-2,6-sialyltransferase I (St6gal1) expression in germinal center B cells and consequently inhibits IgG Fc sialyation (288). Furthermore, IL-23-activation of T_h_17 cells drives decreased Fc sialyation via IL-21 and IL-22-dependent downregulation of St6gal1 expression in plasmablasts and plasma cells (289).

Given the direct role of IgG Fc glycosylation in Fc effector functions which are both influenced and regulated by inflammation (264), glycosylation has been identified as a useful biomarker of chronic and acute inflammation as well as disease progression and severity in the context of both infectious and noncommunicable diseases (110, 111, 290–297). IgG afucosylation is a pro-inflammatory hallmark, owing largely to ADCC upregulation (296). Afucosylation is associated with heightened COVID-19 and dengue fever severity owing to the excessive inflammation to which afucosylated IgG contributes (110, 111, 290, 292, 296). However, in the setting of chronic infection, upregulated effector functions may be a protective adaptation enabling relatively slower disease progression. As such, reduced fucose abundance is associated with favourable disease outcomes, contributing to HIV-1 control and tuberculosis (TB) latency (53, 79). Whether increased abundance of fucosylated IgG is ultimately pathogenic or protective is highly disease specific and is underpinned by whether enhanced ADCC can promote pathogen clearance without inducing detrimental hyperinflammatory responses.

Reduced IgG galactosylation during chronic infection may be beneficial or detrimental to disease control depending upon the protective capacity of the upregulated Fc effector functions in the specific disease context (271–274). Indeed, agalactosylation of both bulk and antigen-specific IgG is associated with spontaneous HIV-1 control (79) as well as longer time to viral rebound following cessation of antiretroviral therapy (298), while increased IgG galactosylation is associated with tuberculosis latency (53). On the other hand, galactose is a key biomarker for the progression of non-communicable inflammatory diseases (297). Increased galactosylation of total IgG is generally associated with improved metabolic health (299, 300), while increased total IgG agalactosylation is associated with progression of inflammatory and autoimmune diseases such as rheumatoid arthritis (297, 301) and systemic lupus erythematous (302). Although this observation appears somewhat counterintuitive given the role of galactose in enhancing inflammatory processes such as ADCD and ADCC, it has been hypothesised that discrepancies in total compared to antigen-specific glycosylation may mediate this effect (303). When global IgG agalactosylation is high, thereby impairing general FcγR engagement, this environment would favour enhanced C1q engagement and FcγR activation by more highly galactosylated antigen-specific autoimmune antibodies with a consequently increased affinity for FcγRs. When global IgG agalactosylation is low, total IgG outcompetes autoantigen-specific autoimmune antibodies for FcγR binding, thereby increasing the threshold required for immune activation by pathologic antibodies (303). In addition, via a separate FcγR-mediated mechanism, terminal galactosylation of IgG1 immune complexes mediates anti-inflammatory activity by promoting FcγRIIb driven inhibition of complement-dependent inflammatory pathways (304).

IgG glycosylation is central to maintaining the fine balance between induction of protective and pathogenic Fc functions, highlighting a critical immunomodulatory role for Fc glycosylation in control of infectious disease, but also the regulatory influence of inflammation upon Fc glycosylation. Indeed, post-translational glycosylation is dynamic and highly sensitive to changes within the B cell microenvironment (288, 305, 306), and as such, may undergo relatively rapid modification dependent upon hormonal (307), vaccine or pathogen-derived stimuli (287, 308), as well as more gradual changes associated with ageing and disease (299, 309).

## Dysregulated Fc effector functions characterise vulnerable populations

Priority populations can be defined by key host factors that influence the vaccine response, including age, sex, immunogenetics, pregnancy, chronic comorbidities, and malignancies (1, 4, 310–313). These clinical and demographic features are further associated with changes to well-characterised and emerging molecular predictors of vaccine-induced protection (22, 314). Some of these predictive biomarkers are highly linked to lifestyle and health status, such as baseline host inflammation and the gut microbiota (315, 316). Other features are more closely tied to age and genetics, such as pre-existing immunity as a result of prior antigen exposure, immune cell frequencies and activation, antibody titre and function, and capacity for antigen processing (37, 317–326). Notably, characteristic modulation of these host variables results in distinct vaccine responses within specific populations (22). Consequently, tailoring vaccine design to elicit the precise immune features lacking in target populations may prove essential for enhancing vaccine effectiveness.

**Figure 5:**
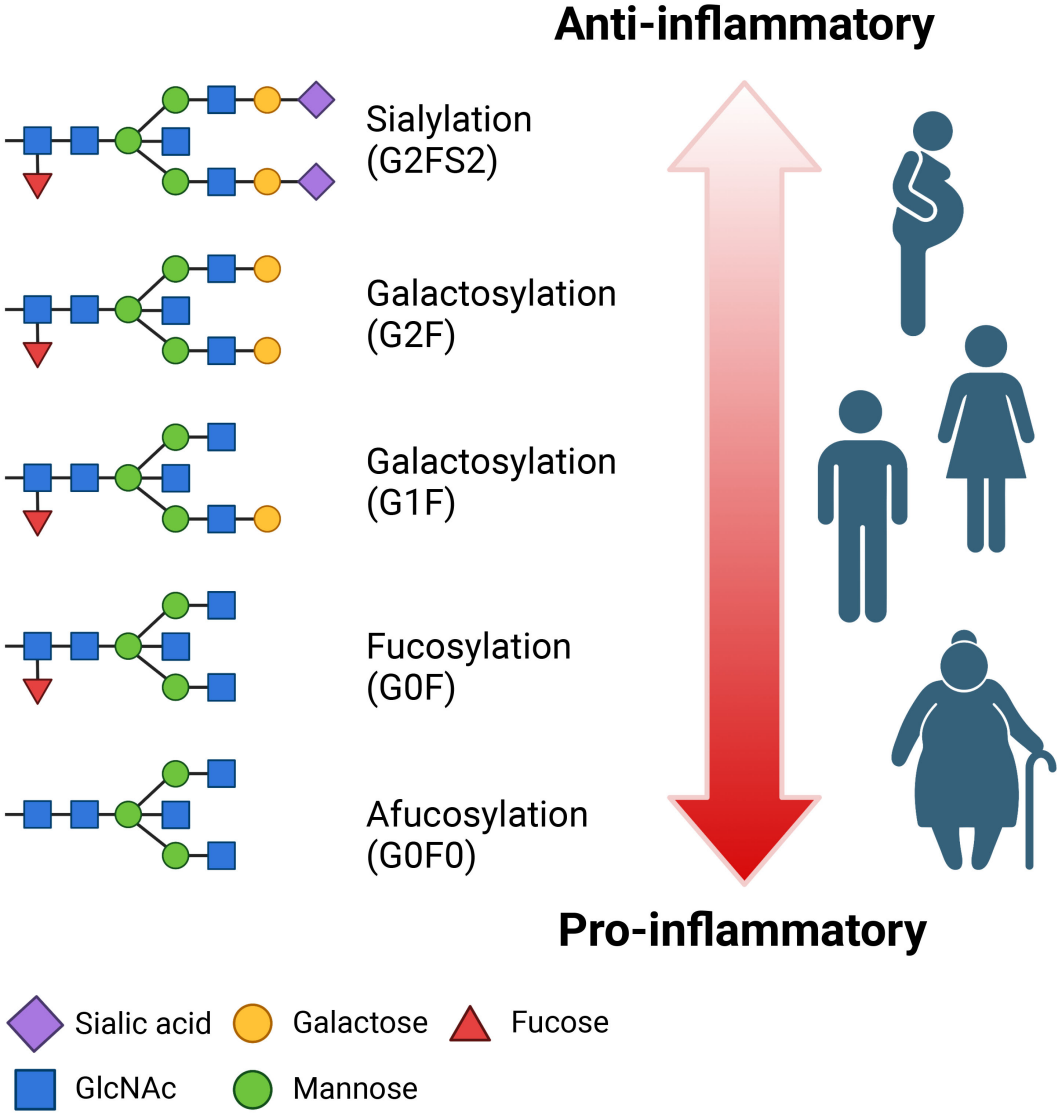
IgG Fc glycan structures have variable inflammatory properties. IgG Fc glycans differentially modulate Fc effector functions and, therefore, inflammation, depending on the interactions of the sugars with various Fc receptors and complement proteins. In general, lack of fucose is highly inflammatory while the presence of galactose and sialic acid is anti-inflammatory. Total IgG Fc glycosylation varies considerably with age, sex, and health status. In general, there is a greater abundance of pro-inflammatory Fc glycans in elderly individuals with chronic comorbidities, such as obesity, and this is particularly elevated in post-menopausal women. On the other end of the spectrum, pregnancy is associated with increased abundance of anti-inflammatory Fc glycans. Among healthy young adults, women typically have a slightly more anti-inflammatory Fc glycan profile.

The underlying mechanisms of immune dysregulation observed in immunocompromised populations is an active area of investigation. However, a perturbed cytokine milieu appears to be central to impaired vaccine responses (327, 328). Notably, many of these immunologically vulnerable groups, including the elderly, individuals with chronic inflammatory conditions and autoimmune disorders, as well as cancer patients, share characteristic patterns of cytokine dysregulation related to imbalances in CD4^+^ T cell subsets (329–334), immunoglobulin class switching (323), and IgG glycosylation (299, 309, 335), both between and within groups (**Figure 5** illustrates IgG glycosylation-specific population trends). As cytokines secreted by CD4^+^ T cells are important B cell stimuli for the regulation of both class switching (336–338) and IgG glycosylation (305)—features which heavily influence Fc functions—a perturbed baseline cytokine milieu may drive Fc effector function dysregulation.

Although poor vaccine immunogenicity in vulnerable populations may, in some instances, be restored by additional vaccine doses (15), boosting is not a universally effective strategy for all vaccines and across all immunocompromised groups (339). Furthermore, modelling suggests that the benefits of boosting may be transient for some immunosuppressed individuals (340). Therefore, in order to design more effective vaccines for immunocompromised groups, a deep understanding of the dysregulated immune networks characteristic of these populations, as well as how these altered immune responses are influenced by different vaccination strategies, is required. Recent systems serology studies have highlighted differences in Fc functions between young, healthy, non-pregnant adults and various vulnerable populations including, children, pregnant women, elderly individuals and patients with various co-morbidities (37, 310, 324, 341–343). Importantly, identification of shared characteristic immunomodulatory mechanisms underlying impaired protection across multiple immunocompromised groups (328, 330, 341) may enable design of more broadly generalised first-generation population-specific vaccine modifications.

### Pregnant women

During pregnancy, to ensure the developing foetus is not rejected, the body maintains a precisely modulated immunosuppressive, anti-inflammatory state that is reflected in generally diminished Fc functions (310, 344) underpinned by a global decrease in inflammatory glycan structures (344, 345). Distinct Fc effector functions have been observed in pregnant and lactating women compared to healthy controls following prime-boost SARS-CoV-2 vaccination, despite equivalent vaccine-specific antibody titres post-boost (310). Pregnant and lactating women displayed delayed Fc kinetics, requiring two doses to generate responses that were comparable, though still reduced, to nonpregnant controls (310). In contrast, post-boost, ADNKA and ADNP trended higher in lactating women than in both pregnant and nonpregnant women (310). Varied functional antibody responses have also been described during pregnancy following influenza vaccination. Compared to their non-pregnant counterparts, pregnant women demonstrated impaired overall Fc function driven by reduced capacity for ADCP and ADCD, which was linked to an increase in anti-inflammatory Fc fucosylation and sialyation (344). Nevertheless, increased galactosylation of both bulk and vaccine-specific Fc antibodies was correlated with improved ADNKA in pregnant compared to non-pregnant influenza vaccinated women (344). Finally, the timing of maternal vaccination may impact Fc-mediated protection, with trends of higher functional antibody responses induced by third trimester SARS-CoV-2 vaccination, followed by first then second trimester vaccination (346).

Chronic infection may further influence pregnancy-induced differences in Fc capacity. For example, pregnancy during HIV-1 infection creates a complex environment of opposing immunomodulatory mechanisms (347). Pregnancy-driven immunosuppression competes with HIV-1 associated chronic inflammation thereby driving a unique IgG Fc glycan profile of decreased galactosylation in pregnant women living with HIV-1 (WLWH) (348). Influenza vaccine-induced Fc effector functions are variably regulated in pregnant WLWH compared to HIV-1-uninfected women (349). Following vaccination, ADCP boosting was evident in otherwise healthy pregnant women but not in pregnant WLWH; ADCD was boosted in both groups but was significantly higher in uninfected women (349). Altogether, these differences in Fc effector capacities may point to baseline IgG glycosylation impacting post-vaccination antigen-specific Fc glycoforms, and therefore, effector functions. These studies suggest there may be value in further tailoring vaccination strategies for vulnerable populations who fall into more than one risk group given the marked effect of highly nuanced baseline inflammation on Fc effector functions.

### Neonates and infants

The health of neonates and infants is inextricably linked to that of the mother (350–353). As such, pregnancy is a unique window during which maternal and infant health can simultaneously be benefitted by a single course of vaccination (354–357). Placental transfer of maternal antibodies is a key mechanism of neonate protection against numerous infectious diseases, including RSV, influenza, pertussis, measles, and tetanus (358, 359). However, studies of HIV-1, malaria, and SARS-CoV-2 infected pregnant women have revealed that placental transfer of related and unrelated antibodies can be compromised by maternal infection (360–364). This outcome may partially explain the increased childhood disease susceptibility of HIV-1 exposed but uninfected infants as well as infants affected by placental malaria (365, 366). Critically, altered IgG subclass distribution and Fc glycosylation has been implicated in the mechanism of impaired placental transfer of antibodies generated both during and prior to infection (361, 367).

In healthy pregnant women, digalactosylated Fc functional antibodies are preferentially transferred during the gestational period in contrast to antibodies lacking the capacity to bind FcRn, FcγRIIa, and FcγRIIIa (358, 359). Most notably, there is preferential transfer of ADNKA capacity to neonates correlating with enhanced binding of digalactosylated IgG1 to FcRn and FcγRIIIa (358). In contrast, ADCP functionality is retained by the mother (358). Furthermore, equivalent antibody Fc functional capacity has been demonstrated in preterm and full term neonates with robust early transfer of ADNKA capacity (359). This early selective sieving of Fc functional capacity, ADNKA, likely points to an evolutionary advantage of increased Fc capacity in early life (358, 359). Indeed, placentally transferred NK cell activating antibodies drive elevated cytokine release by umbilical cord NK cells compared to adult NK cells (358).

The nature of this placental sieve has implications for the rational design and timing of vaccines administered to pregnant women. For example, vaccine regimens that elicit highly galactosylated antibodies with enhanced affinity for FcRn may be more efficiently transferred and, therefore, afford elevated neonate protection. Indeed, increased placental transfer efficacy of Fc functional SARS-CoV-2 specific antibodies has been observed following mRNA-1273 or BN162b2 lipid nanoparticle mRNA vaccination compared to Ad26.COV2.S adenoviral vector vaccination, with further subtle increases elicited by mRNA-1273 compared to BN162b2 vaccination (346). This suggests that vaccine formulation may substantially alter the functional capacity of antibodies transferred to neonates. On the other hand, maternal antibodies may limit humoral responses in infants following vaccination (368). Although the mechanism remains contested, epitope masking and inhibitory FcγRIIb engagement by maternal antibodies may contribute to this outcome (369, 370). Given that different epitopes drive differential Fc functions (371), immunogen selection for maternal vaccines should also consider the possible impacts on early childhood vaccine responses.

### Children

Children under five are highly susceptible to infectious diseases. Numerous cellular and humoral deficiencies define the immature immune system, however, altered antibody class switching (220, 221) and IgG glycosylation (372, 373) confer young children unique Fc effector profiles. Rational vaccine design which exploits the elevated Fc capacity (37, 324, 374, 375) of childhood humoral immunity may promote optimised protection.

While age-related variation in IgG glycosylation is well-recognised (309, 335, 376), detailed data from paediatric cohorts is limited. Nevertheless, evidence exists for variation across childhood and adolescence, with an overall trend of decreased inflammatory agalactosylation with increasing age (372, 373, 376–378). However, further dissection of IgG glycosylation patterns in the first two years of life has revealed increased anti-inflammatory IgG glycoforms with increased digalactosylation, sialyation, and core fucosylation in children aged 9 months to 2 years compared to older children up to 5 years. Between ages 2 to 5 years, IgG glycosylation shifts to a more pro-inflammatory profile of increased agalactosylation and reduced sialyation, before the production of increasingly galactosylated IgG commences (372, 373). Notably, IgG glycosylation patterns have been identified as a potential biomarker of recurrent respiratory infections (RRI) in childhood (372). Interestingly, increased anti-inflammatory digalactosylated and sialyated IgG were enriched in the RRI group—suggesting that decreased effector potency of these antibodies could leave children more vulnerable to repeated infections.

Increased class switching to more mature IgG2 and IgG4 isotypes gradually occurs from infancy to adolescence (220). As such, the baseline production of increased levels of IgG1 and IgG3 in young children under 3 years may be advantageous for the generation of highly Fc functional antibodies by early childhood vaccines. Indeed, children develop elevated IgG1 titres and enhanced Fc functional responses, including ADNP and ADCD, as well as FcγRIIIa binding upon SARS-CoV-2 vaccination in comparison to adults (324). This increase in Fc functional capacity was especially evident when children were administered the full adult dose of Moderna mRNA-1273 vaccine, rather than the reduced paediatric dose (324), underscoring the impact of vaccine dosage on Fc functions.

Robust Fc effector functions in antiretroviral therapy-naïve HIV-1 infected children have also been observed (379, 380), and are especially elevated in paediatric HIV-1 non-progressors (PNP; i.e., children who maintain normal CD4^+^ T cell counts despite ongoing high viral replication in the absence of antiretroviral therapy) compared to in progressors (380). ADNKA, likely driven by robust IgG1 responses, is consistently observed across cohorts (379, 380), and, along with decreased Fc fucosylation, may contribute to disease control in PNP (380). Notably, coordination of Fc effector responses (379) and increased antigen-specific IgG Fc sialylation (380) were positively associated with neutralisation breadth, suggesting dual benefit to vaccines targeting the generation of Fc functional antibodies.

### Elderly individuals

The ageing humoral immune system is characterised by immunosenescence induced by both chronic low-grade inflammation and prior antigen exposure leading to reduced antibody titres and largely diminished vaccine responses (327, 334, 381). The resultant upregulation of inflammatory cytokines such as IL-6, IL-1β, tumour necrosis factor α, as well as decreased anti-inflammatory cytokines such as IL-10, may contribute to impairments across a broad range of humoral immune system features, including B cell activation, antibody class switching, affinity maturation, and Fc glycosylation (300, 382–384). Reduced expression of AID, associated with transcription factor E47 downregulation, is suggested to dampen capacity for CSR, as reflected by the diminished pool of switched memory B cells in elderly individuals (385). Consequently, class switching to cytophilic IgG1 and IgG3, may be diminished in elderly individuals (237, 238, 386, 387). Increased age is also associated with increased baseline abundance of pro-inflammatory agalactosylated and asialylated IgG (300, 309, 335, 376) which may contribute to generation of dampened or uncoordinated Fc effector functions upon vaccination. Overall, these antibody impairments likely underpin the decreased FcγR binding and Fc effector functions observed in elderly individuals (37, 388).

Beyond the current approach of early and additional vaccine doses for elderly individuals, a combination of more targeted strategies may benefit this population (13, 15, 389). In the case of influenza, poor vaccine immunogenicity in the elderly may be partially overcome by high-dose vaccination (390, 391) and inclusion of adjuvants such as MF59 (391, 392) and AS03 (393). However, this population may further benefit from vaccines specifically formulated to elicit potent Fc effector functions upon a background of dysregulated IgG class switching and Fc glycosylation. Although MF59 selectively boosts IgG3 titres and may bolster generation of Fc functional antibodies (394) when class switching is impaired, eliciting Fc glycosylation patterns that support enhanced FcγR engagement may further improve vaccine effectiveness. Notably, the influence of age upon FcγR engagement and effector functions following SARS-CoV-2 vaccination is conflicting, with studies reporting positive (395), negative (396), and no association (50). However, these trends were determined via small to moderately sized patient cohorts, underscoring the need for larger clinical trials to adequately address this critical question.

### Patients with chronic comorbidities

Many non-communicable diseases associated with chronic low-grade inflammation have increased in prevalence in recent decades, particularly in high- and middle-income countries (397–399). This phenomenon may reduce effectiveness of vaccines which are typically less immunogenic in patients experiencing chronic inflammation as a result of malignancies, autoimmune diseases, and obesity. Furthermore, both immunosuppressant drugs—used to mitigate symptoms of inflammatory autoimmune diseases and manage solid organ transplants (400)—as well as the chemotherapy and radiation regimens—used to treat malignancies—may render vaccines less immunogenic.

Rheumatoid arthritis, systemic lupus erythematous, renal disease, and inflammatory bowel disease as well as other chronic conditions associated with dysregulated inflammation, such as obesity and type 2 diabetes, contribute substantially to the global burden of comorbidities that reduce vaccine effectiveness (401). Most notably, research investigating vaccine responses in obese patients has revealed a proinflammatory cytokine milieu associated with a dysregulated humoral response, similar to that observed in elderly individuals (330, 332, 402, 403). Impaired humoral immunity upon vaccination is most readily evidenced by reduced antibody titres and neutralisation capacity (311, 404, 405). However, Fc effector capacity in these populations may also be highly dysregulated, largely driven by aberrant IgG Fc glycosylation underpinned by increased IgG Fc agalactosylation, asialyation, and afucosylation (299, 302).

Large networks of genes which regulate Fc glycosylation are pleiotropic with inflammatory diseases such as rheumatoid arthritis and inflammatory bowel disease (406). However, it has long been appreciated that increased pro-inflammatory agalactosylation is a biomarker of disease onset and severity for many of these conditions (301, 302, 377). Increased proinflammatory IgG glycosylation has also been defined for a variety of malignancies, including multiple myeloma (407), colorectal cancer (CRC) (408, 409), thyroid cancer (335), and ovarian cancer (410). Notably, in a study of patients receiving allogeneic hematopoietic stem cell transplantation, post-procedural recipient IgG glycosylation more closely resembled their pre-transplantation profiles than that of donor IgG glycosylation (411). This reinforces the predominant role for the B cell microenvironment in driving IgG glycosylation patterns and suggests that the persistence of patient-specific immunomodulation such as hormone dysregulation, CD4^+^ T cell perturbances, and inflammatory cytokines may have long-term consequences for the vaccination of patients with haematological malignancies.

Although likely dysregulated, Fc functions appear to be better preserved than neutralisation capacity in immunosuppressed populations. In a study of SARS-CoV-2 vaccination of cancer patients, anti-spike antibody titres were generally concordant with neutralising titres against the wild-type virus (405). However, this trend was not observed against variants of concern where over half of individuals generating anti-spike antibody responses were unable to neutralise SARS-CoV-2 variants (405). Although alterations to IgG Fc glycosylation and effector functions are heavily studied in the context of tumour clearance and cancer progression and survival (269, 412, 413), the effect of these malignancy-induced modifications upon immune responses to vaccination and infection remains understudied.

As IgG Fc glycosylation contributes substantially to Fc effector function potency, designing vaccines that counter perturbed IgG Fc glycosylation patterns and elicit coordinated Fc functions may enhance protective responses in populations experiencing dysregulated inflammation. Indeed, pro-inflammatory IgG glycan abundance has been associated with impaired influenza (414) and SARS-CoV-2 (395) vaccination. Increased baseline level of agalactosylated total IgG was a signature of influenza vaccine non-responders, while elevated IgG galactose abundance predicted robust vaccine response (414). Similarly, elevated baseline abundance of anti-inflammatory galactosylated, sialylated, and fucosylated IgG1 correlated with higher anti-SARS-CoV-2 IgG titres following vaccination (395).

Finally, dysbiosis of the gut microbiome is frequent in obese individuals, as well as patients with malignancies and chronic inflammatory conditions (401, 415–421). There is an established role for the gut microbiome in regulating antibody titres following vaccination (315, 316, 422). Hence, it is plausible that gut dysbiosis may also impair Fc effector functions by modulating inflammatory cytokine levels and subsequently influencing IgG Fc glycosylation and downstream effector functions.

## Modulation of Fc effector functions in healthy adult populations

Distinct groups of healthy individuals may also benefit from population-based vaccination strategies. Biological sex-specific differences can impact both antibody quantity and quality, with age-dependent variation in glycosylation patterns (423) likely influencing Fc functional responses (37). Immunogenetics further impact functional antibody responses via allotype associated variations in IgG subclass distribution and FcγR polymorphisms that alter affinity for IgG. Finally, the gut microbiome within healthy individuals may also influence Fc functions by promoting inflammatory processes that modulate IgG glycosylation.

### Sex-based differences in vaccine responses

Across age groups, females typically generate more robust humoral responses to many vaccines than do males, with higher antibody titres observed following vaccination against influenza, HBV, yellow fever virus, dengue virus, and measles, mumps and rubella (424–426). However, females may generate a more functional antibody response with increased class switching to IgG3 directing more robust Fc effector functions against some pathogens (427) while males may generate increased titres of poorly functional IgG4 (428). Furthermore, young to middle-aged women typically have increased abundance of anti-inflammatory galactosylated IgG than their male counterparts. However, elderly women have increased abundance of agalactosylated IgG—a phenomenon associated with onset of menopause, likely owing to reduced estrogen levels (307, 309, 429). In addition, females typically have increased phagocytic effector cell frequencies while males have higher NK cell counts but with decreased effector capacity compared to females (430, 431). Differences in IgG Fc glycosylation and innate cell frequencies result in nuanced differences in effector functions between the sexes. For example, males typically generate more robust ADCC in the context of measles (432) or HIV-1 infection (433). As such, men and women may benefit differently from vaccination regimens that aim to either elevate antibody titre or enhance FcγR engagement. Males may benefit from inclusion of adjuvants that enhance class switching to IgG3 (e.g. MF59). On the other hand, given heightened vaccine immunogenicity and reactogenicity, females may benefit from reduced dose regimens that elicit more coordinated Fc functions and fewer adverse effects.

### Immunogenetics

Polymorphisms within *IGHG* and *FCGR* genes, as well as *FCGR* copy number variations, are associated with differential responses to infection and vaccination for a range of pathogens (**Supplementary Table**). The potential for IgG allotypes to modulate Fc functions is largely driven by the altered subclass distributions associated with different haplotypes, and to a much lesser extent, the altered FcγRIIIa affinity of specific allotypes, as previously discussed (255). On the other hand, FcγR polymorphisms influence Fc functions via the increased affinity of FcγRIIa-131H and FcγRIIIa-158V for IgG subclasses (60).

Epistatic interaction of FcγRIIIa polymorphisms and IgG1 allotypes has been observed in HSV-1 infection such that, as a result of enhanced ADCC, the high affinity FcγRIIIa-158V/V genotype was only associated with asymptomatic infection in individuals homozygous for the G1m3 IgG1 allotype (434), typically linked to reduced IgG1 responses against viral infections. This protective effect may not have been observed in G1m17 homozygotes given the increased affinity of G1m17 IgG1 for the HSV-1 decoy FcγR compared to the antithetical G1m3 allotype (435), possibly resulting in increased clearance of G1m17 IgG1. Notably, whether high or low affinity FcγR alleles confer a protective or deleterious effect is likely a disease specific phenomenon, which is presented in detail within the **Supplementary Table**.

In addition, the influence of human leukocyte antigen (*HLA*) alleles has long been understood to impact vaccine outcomes. Given the ethnic clustering of HLA allomorphs, different populations demonstrate varying levels of vaccine-induced protection and disease susceptibility (312, 436–440). Although not directly responsible for shaping the functional antibody response, certain *HLA* allomorphs have been associated with increased antibody titres against SARS-CoV-2 (438), and the potential interaction of *HLA*, immunoglobulin kappa chain, IgG constant region and FcγR polymorphisms cannot be ignored in the design of population-based vaccines informed by immunogenetic features (434, 441–443). The interplay of these genetic polymorphisms is of particular importance in Indigenous populations who are frequently underrepresented in vaccine studies (8, 9) and whose unique immunogenetic backgrounds may underlie differential vaccine responses and infection susceptibility (250, 437, 439).

**Figure 6:**
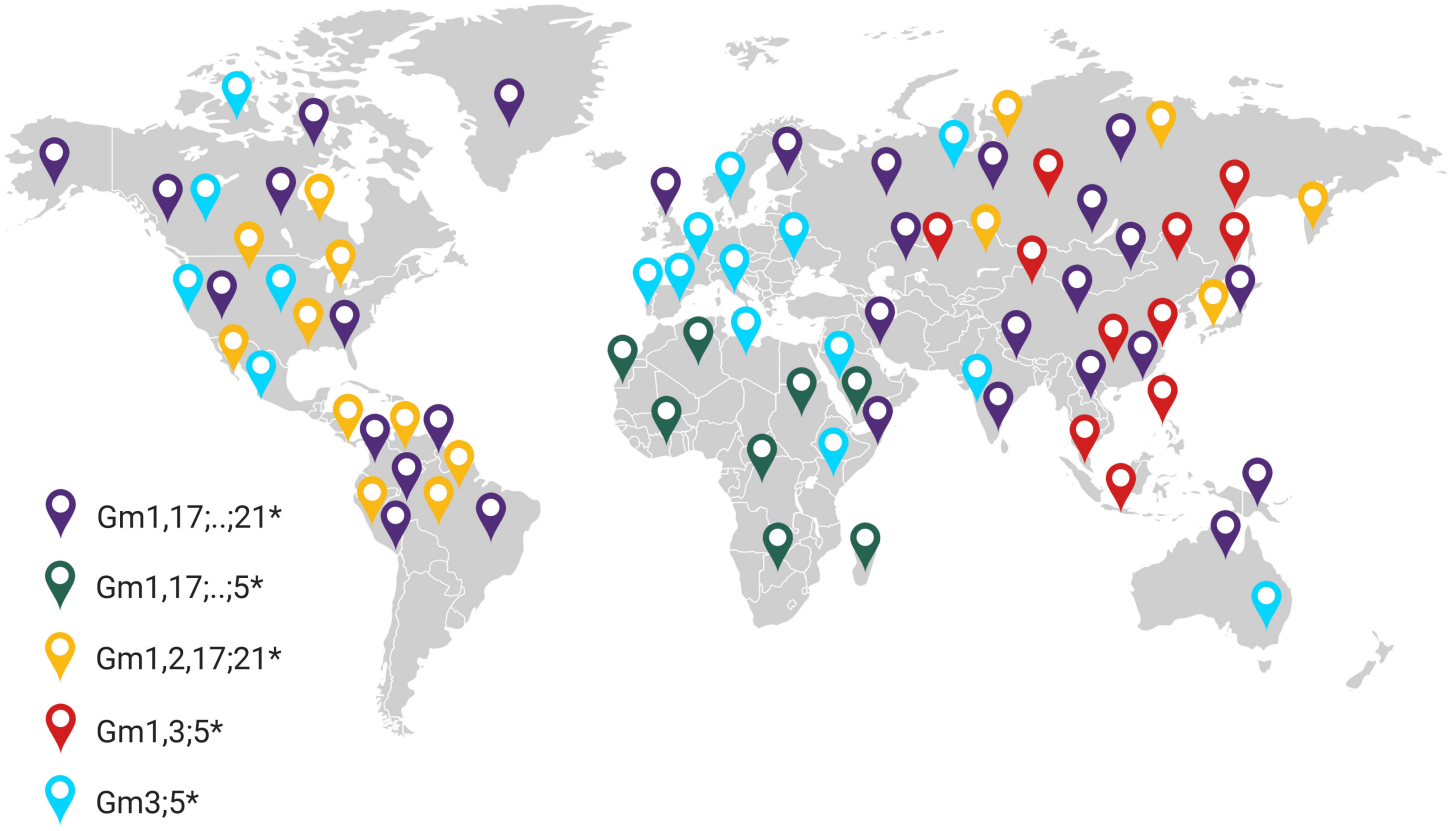
Geographic distribution of dominant IgG haplotypes. IgG allotypes are inherited as haplotype blocks and thus show geographic clustering within ethnicities. Data compiled from (449).

The potential value of considering immunogenetic influences upon vaccine responses has recently emerged through comparisons of analogous HIV-1 vaccine trial efficacies derived from different study populations. The RV144 trial, conducted in Thailand with participants of predominantly South-East Asian ethnicity, demonstrated 31.2% efficacy (81). When the RV144-inspired HVTN702 follow up trial was conducted in South Africa, modified to reflect the dominant circulating HIV-1 subtype, the vaccine showed no efficacy (444). Subsequent computational analyses indicated immunogenetics may have contributed to variable protective outcomes between the trials (445, 446). Host immunogenetic diversity, particularly within the *IGHG* locus varies substantially between the Thai and South African populations (447). Importantly, given *IGHG*, *FCGR*, and *HLA* genotypes show distinct geographic clustering (248, 447, 448), the possibility exists for their influence to be modelled into future population-based vaccines. **Figure 6** illustrates the geographic clustering of dominant IgG haplotypes.

## Vaccination strategies modulate Fc effector functions

There is growing consensus that precise modulation of Fc effector functions is a valuable goal of future vaccines and may be key to optimising vaccine responses in certain populations (445, 450). Dysregulated total IgG subclass ratios and global IgG glycosylation are not altered following vaccination (210, 308, 414). However, antigen-specific IgG subclass distribution and antigen-specific IgG glycosylation—key modulators of Fc effector functions—are tuneable via vaccination (210, 211). Furthermore, vaccination can override differences in baseline IgG glycosylation observed between healthy populations from distinct geographic locations (210, 308). As such, these antibody features are rational targets of vaccines designed to boost Fc effector functions in vulnerable populations.

However, given the challenge of eliciting precisely selected Fc functions, most data indicating vaccination-induced differences in functional antibody responses are derived from serendipitous observations following variations to immunogen, vaccine platform, adjuvant, dosage, and administration route. As such, defining the mechanisms of modulation along with strategies that enable fine-tuning of Fc effector functions via vaccination is of high priority for the precision vaccination field.

### Immunogen selection

Although Fc effector functions can theoretically be initiated upon binding to any epitope, certain epitopes can drive more potent effector functions than others. The influence of Fab specificity upon Fc-FcR interactions and effector functions has recently been demonstrated in studies of influenza, HIV-1, and Ebola (49, 54, 55, 371). Emerging data has also implicated Fab-FcγR interactions in ADCC potency (451). In addition, antigen valency may impact Fc functions in a Fab-specific manner given that FcγR activation requires dimerisation facilitated by cooperation between at least two antibodies. Furthermore, IgG is glycosylated in an antigen-specific manner (70, 213). Different antigens from the same pathogen (210) or even the same protein (452), may induce differential IgG glycosylation which may impact Fc effector functions. As such, carefully informed choice of immunogen is critical to rational vaccine design.

However, immunogen-specific modulation of Fc functions is largely underpinned by the conformational accessibility of different epitopes as both Fab-antigen and Fc-FcR interactions must be simultaneously accommodated. Indeed, angle of approach of certain Fab-antigen interactions may result in steric hinderance of Fc-FcR engagement (56) or allosteric changes to antibody conformation upon binding which promote or impair FcR interactions (453, 454). Epitope proximity to the viral envelope or target cell membrane has been suggested to influence Fc functions (57, 455, 456). However, studies of ADCP in this context are conflicting, with reports of both enhanced (455) and impaired (456) potency with increasing distance from target cells. Nevertheless, studies of mAbs against the Ebola virus surface glycoprotein indicated that antibodies against epitopes farthest from viral envelope were the most polyfunctional (57, 457). These findings resonate with observations across of a variety of antigens that IgG1 and IgG3 hinge length polymorphisms contribute to ADCC and ADCP potency, with increasing hinge length promoting enhanced ADCP (228, 232, 233, 458) but decreased ADCC (255).

In a study of influenza A mAbs, anti-HA stalk-specific antibodies induced ADCC while those targeting the head region antibodies did not (49). However, the authors later demonstrated that this observation was not a broadly generalisable rule and that other anti-HA head antibodies mediate protective Fc functions (154). This suggests that the precise antibody footprint may have a greater influence over ADCC induction than the general epitope location. Nevertheless, a separate study found that across mouse and human mAbs, as well as polyclonal human IgG, anti-HA head antibodies did not induce ADCC and further inhibited anti-stalk mAb directed ADCC (54). Consequently, suggestions have been posited to increase Fc effector function via immunogen design. For example, shielding the immunodominant HA head via glycosylation may bias responses towards the stalk and enhance Fc functional responses (459).

### Vaccine platform

Recent innovations in materials science have expanded licenced vaccine platform options beyond traditional live attenuated, inactivated, recombinant, and viral vector vaccines to include a range of nanoparticle vaccines (28). Of note, the value of lipid nanoparticle mRNA vaccines was demonstrated by their increased effectiveness in comparison to traditional platforms during the COVID-19 pandemic (39, 460). Unique features of each vaccine platform enable varied interactions with the immune system. As such, delivery of the same antigen via different modalities can elicit markedly different responses, including distinct changes to IgG glycosylation and downstream antibody effector functions, which impact vaccine efficacy.

Previous studies have identified an increased abundance of vaccine-specific galactosylated and sialylated IgG following tetanus toxoid and inactivated influenza and vaccination (308, 452). However, comparison of different SARS-CoV-2 vaccines regimens has allowed for more granular dissection of the impacts of vaccine formulation upon IgG glycosylation (212). Pfizer BioNTech BNT162b2 SARS-CoV-2 mRNA vaccination induces an initial transient pattern of increased spike-specific IgG1 Fc galactosylation and sialylation but decreased fucosylation (212, 461). Over time, antigen-specific IgG1 fucosylation levels gradually increased to above that of total IgG1, while galactosylation and sialylation levels gradually decreased, with levels of galactosylated vaccine-specific IgG1 falling below that of total IgG1 by day 190 post-vaccination (212). On the other hand, vaccination with AstraZeneca SARS-CoV-2 AZD1222 adenoviral vector produced a less pronounced decrease in fucosylation immediately post-vaccination, falling to only 95% as compared to the 80% fucosylation induced by BNT162b2 vaccination (212). Similarly, the increase in IgG1 Fc galactosylation was less pronounced after one dose of AZD1222 than BNT162b2 vaccination (212). These kinetics of IgG glycosylation following SARS-CoV-2 mRNA vaccination are in line with previous studies reporting increased antigen-specific IgG fuscoylation in the weeks following vaccination (395, 462), which may promote coordinated Fc effector functions by limiting inflammation (462).

The mechanisms by which varied vaccination platforms induce distinct Fc functions is poorly understood. However, nanomaterial vaccine platforms appear to offer a more defined strategy for enhancing antibody polyfunctionality. Nanomaterials facilitate highly ordered, repetitive antigen array that mimics the immunogenicity of many live pathogens (28). Compared to soluble antigen, the multivalent antigen presentation afforded by nanomaterials drives swift trafficking and concentration within germinal centres (463) as well as rapid B cell activation and differentiation (464). This increased antigen deposition and lymph node expansion facilitating improved B cell and T_fh_ cell responses is associated with generation of higher antibody titres and improved protection against influenza challenge (465, 466). Nevertheless, although B cell stimuli are known to impact IgG glycosylation (305), knowledge gaps remain in our understanding of the mechanism by which nanomaterials alter IgG Fc glycosylation and effector functions.

Recently, a nanomaterial-based HIV-1 vaccine was demonstrated to induce potent Fc effector functions correlating with unique antigen-specific antibody glycosylation (467). Q11—a frequently utilised vaccine nanomaterial—was conjugated to gp120, and co-administered with the Fc effector function enhancing adjuvant STR8S-C. This combination stimulated increased neutralisation and antibody breadth, as well as enhanced ADCC and, to a lesser degree, ADCP in rabbits (467). These enhancements to ADCC were correlated with changes in IgG Fc glycosylation patterns, including increased fucosylation and monogalactosylation of gp120-specific antibodies. Furthermore, similar glycan profiles were observed for both mice and rabbits vaccinated with or without the STR8S-C adjuvant. Altogether, these findings suggest that while the impact of STR8S-C adjuvant on glycosylation could not be excluded, the Q11 nanofiber itself was responsible for Fc glycosylation modifications. Afucsoyation of IgG Fc glycans is one of the best-defined divers of increased engagement with ADCC-mediating FcγRIIIa (266–268). That the Q11-gp120 vaccine elicited robust ADCC regardless of overall fucose abundance (467) raises important questions regarding the effects of specific combinations of Fc glycans upon antibody-dependent effector functions and warrants further investigation.

There is emerging appreciation of the role of Fc glycosylation in enhancing neutralisation breadth and affinity maturation (380, 468). The Q11-gp120 vaccine induced both increased IgG Fc sialyation and increased Fab binding breadth (467). Increased sialyation has previously been shown to enhance HIV-1 antigen-antibody complex deposition which in turn was associated with increased neutralising antibody breadth, suggesting that specific Fc glycosylation patterns may impact affinity maturation (380, 468). Fc sialylated immune complexes have similarly been shown to enhance affinity maturation and breadth of anti-influenza antibodies (452, 469). Mechanistically, this is explained by increased binding of sialylated immune complexes to inhibitory FcγRIIb, thereby elevating the B cell receptor threshold of activation (452, 469). As such, it is possible that the improved binding breadth generated by Q11-gp120 vaccination was a consequence of increased sialylation. This would suggest dual benefit to precise modulation of IgG glycosylation for the generation of robust polyfunctional antibody responses: enhanced Fc effector functions and increased Fab binding breadth.

The HIV-1 vaccine trials VAX003 and B003/IPCAVD-004/HVTN 091 comprising vaccines based upon recombinant protein subunit and adenoviral vector systems, respectively, induced differently glycosylated IgG against gp120—a key HIV-1 viral entry envelope glycoprotein (210). The VAX003 regimen induced a more inflammatory response of decreased IgG sialylation and galactosylation compared to that raised by B003/IPCAVD-004/HVTN 091 participants (210). VAX003 consisted of 7 doses of recombinant gp120 protein which resulted in reduced IgG3 titres but enhanced IgG4 titres that inhibited both ADCC and ADCP, potentially contributing to the inefficacy of VAX003 (82, 83). In comparison, the moderately protective RV144 vaccine regimen consisted of a canarypox vector prime followed by only two doses of the same gp120 recombinant protein boost used in VAX003 and was associated with increased antigen-specific IgG3, which was identified as a correlated of protection (82, 83). Intriguingly, low IgG4 levels, similar to that observed in RV144 vaccinees, were observed after only two doses of VAX003, suggesting that repeated protein boosting may have contributed to skewed IgG4 subclass profile (82). On the other hand, the zoster vaccine, consisting of two doses of adjuvanted recombinant glycoprotein E elicited improved ADCC against herpes zoster compared to the live virus vaccine (388). This suggests that muted functional responses are not necessarily inherent to recombinant protein vaccine platforms and are likely also influenced by number of doses.

Interestingly, repeated mRNA SARS-CoV-2 vaccination with either Pfizer BioNTech BNT162b2 or Moderna mRNA-1273 has been shown to induce elevated titres of noninflammatory IgG2 and IgG4 against the viral spike protein (211, 212). Six months following second dose vaccination, in a cohort of 29 individuals, IgG4 increased from 0.04% to 4.82%, which further increased to 19.27% six months following third dose vaccination. Increased IgG4 correlated with increased avidity. However, in line with the non-inflammatory properties of IgG4, this shift in subclass distribution hindered Fc effector functions, with significantly decreased ADCP and ADCD observed following the third compared to the second mRNA vaccine dose (211). In contrast, this phenomenon of elevated IgG4 induction was not observed for adenoviral based SARS-CoV-2 vaccines (212). However, primary two-dose mRNA-1273 vaccination followed by Novavax NVX-CoV2373 recombinant protein nanoparticle booster also promoted elevated IgG4 titres in rhesus macaques (470). Whether the increase in IgG4 and associated decrease in Fc effector functions reduces protection or is beneficial to mitigating potential ADCC-driven immunopathology following SARS-CoV-2 infection remains to be determined (211).

### Dosing quantity and schedule

The magnitude and timing of vaccine doses can also vastly impact the quality and quantity of antibody responses. For example, two highly similar vaccination platforms delivered in an altered format and dose (Moderna mRNA-1273 and Pfizer BioNTech BNT162b2 SARS-CoV-2 mRNA lipid nanoparticle vaccines) have recently been shown to yield differential functional responses (471). Compared to BNT162b2, mRNA-1273 vaccination induced higher levels of ADNP and ADNKA (471). It has been suggested that the increased dosing interval of the mRNA-1273 vaccine regimen allowed for a more coordinated functional response to develop (471). Likewise, a subsequent study observed that an increased BNT162b2 dosing interval was also associated with enhanced vaccine immunogenicity (340). However, it is possible that the greater mRNA-1273 antigen dose or lipid nanoparticle formulation and mRNA modifications specific to each vaccine may additionally contribute to generation of superior Fc effector capacities via mRNA-1273 vaccination. Nevertheless, this study highlights the potential for fine-tuning Fc effector functions via precision vaccination strategies.

Characterisation of the impact of SARS-CoV-2 adenoviral and protein subunit vaccine dosage has provided more detailed evidence that the quality and durability of antibody Fc effector functions is regulated by antigen quantity per exposure. However, these trends do not appear consistent across different vaccine platforms (78, 204). A nonhuman primate study of the dose-dependent effects of Johnson & Johnson Ad26.CoV2.S adenoviral vector vaccination observed that increased FcγR receptor binding and Fc functional antibody levels, trended strongly with increased dosage, whereas neutralising antibody titres and T cell responses were minimally affected (204). In contrast, a second nonhuman primate study using the Novavax NVX-CoV2373 recombinant SARS-CoV-2 spike protein nanoparticle vaccine with Matrix M adjuvant demonstrated that increased ADNP and ADNKA were associated with the administration of a lower antigen dose (78). Importantly, lower ADCP, ADNP, and ADNKA were observed within single dose groups as compared to their two dose counterparts (78). As such, a single NVX-CoV2373 dose provided only partial protection, in contrast to the near-complete protection of two doses associated with marked maturation of Fc effector functions. In addition, although long priming of germinal centres with sustained antigen delivery in escalating dose vaccination strategies has been shown to improve antibody titres and affinity (472, 473), investigation into the impact of escalating dosage upon Fc effector functions is warranted.

A systems serology analysis identified that delayed fractional dosing of the RTS,S/AS01 vaccine in controlled human malaria infection models increased Fc polyfunctionality (69). Qualitative enhancement of both the Fab and Fc in vaccinees was underpinned by increased ADCP, ADNP, and antibody-dependent dendritic cell phagocytosis (ADDCP), particularly against subdominant epitopes, and correlated with increased Fab region avidity (69). Importantly, this malaria vaccine regimen maintained the immunodominant NANP6 region-specific ADCP and ADNKA (69) which were previously defined as correlates of protection for the standard vaccination schedule (92). However, a separate study associated delayed fractional dosing of the RTS,S/AS01 regimen with increased IgG4 titres that inhibited phagocytosis (474). Although delayed fractional dosing increased vaccine efficacy in malaria naïve adults (475), the efficacy of this regimen in malaria exposed populations for whom the vaccine is most relevant remains controversial (476). Nevertheless, the substantially different Fc functions induced by different dosage and timing of the RTS,S/AS01 vaccine (69) reiterate the importance of both antigen quantity and dosing interval in driving an optimised Fc response.

In addition to the effects of antigen dosage within a single vaccine regimen, prior antigen exposure as a result of infection or vaccination may further influence the magnitude, breadth, and function of post-vaccination antibody responses via several mechanisms (477). Prior antigen exposure can induce immune imprinting which can restrict *de novo* immune responses to antigens related to those previously encountered (478). Furthermore, increased pre-vaccination antibody titres may drive accelerated clearance of immune complexes and a decreased window of vaccine antigen presentation (478). However, the presence of pre-existing antibodies can also be beneficial. Influenza vaccination studies have observed that elevated baseline FcγRIIb binding, along with elevated pre-existing IgG2 and decreased pre-existing IgM levels, are associated with increased neutralisation breadth (479). It has been suggested that this may result from decreased immune complex clearance owing to poor FcγR engagement by IgG2, as well as enhanced antigen presentation on follicular dendritic cells via FcγRIIb-binding antibodies. In addition, engagement with inhibitory FcγRIIb on B cells may increase the threshold of activation, thereby driving selection of higher affinity antibodies (480).

Finally, in early childhood vaccines, the timing of initial vaccination may play a role in functional antibody durability. The long-term functional capacity of measles-specific antibodies appeared to be more durable if children were vaccinated at 14 months compared to those vaccination between 6-8 months (160). Despite similar functional antibody responses in both groups one-year post-vaccination, children vaccinated later in life had more robust anti-measles functional responses at three years post-vaccination (160). No variation in isotypes or IgG subclasses were observed between age groups, suggesting that other mechanisms of Fc modulation may have contributed to functional differences. For example, variable B cell programming during different stages of early childhood may have led to enduring differences in IgG glycosylation patterns (160), and should be considered when designing childhood immunisation schedules. Alternatively, variable epitope selection driven by waning interference from maternal antibodies with increasing age (369, 370) may have contributed to differential Fc functional responses.

### Adjuvants

A wide range of adjuvants that enhance vaccine immunogenicity—via diverse mechanisms leading to distinct immunological profiles—are approved for human use or in trial (481). This may be highly advantageous for the design of precision vaccines tailored to the unique requirements of distinct immunologically vulnerable populations. Most notably, emulsion adjuvants, such as MF59 and AS03, as well as toll-like receptor (TLR) agonist adjuvants, particularly when used in combination, have been advantageous for generating polyfunctional antibodies and further boosting Fc functional capacity of nanoparticle-based vaccines (467, 482, 483). Nevertheless, comprehensive studies that systematically compare the impact of a spectrum of adjuvants upon Fc functions are lacking. However, systems serology has reiterated the value of TLR agonist-based and emulsion adjuvants for enhancing Fc effector functions (484, 485).

Various TLR agonists that drive differential IgG glycosylation are employed as adjuvants. Macaque studies of simian immunodeficiency virus (SIV) vaccination have shown that distinct TLR agonist adjuvant combinations induce unique antibody Fc functions (486). Further studies have defined a role for differential Fc glycosylation in the modulation of Fc functions by TLR agonists (487). Although different adjuvants induced equivalent protective antibody responses of similar magnitudes, quantitative antibody differences were evident between the groups. A TLR4 plus TLR7 agonist (glucopyranosyl lipid plus imiquimod) adjuvant system stimulated increased ADNP and ADCC, while the TLR4 agonist plus the saponin-derivative adjuvant QS21 was associated with ADCD and anti-inflammatory digalactosylated monosialylated IgG. Although the healthy macaques were equivalently protected, differentially induced Fc effector functions may be of value to vulnerable human vaccinees experiencing dysregulated baseline inflammation. Dissecting which qualitative antibody features may have the greatest protective capacity in this context remains to be determined.

MF59—a squalene oil and surfactant adjuvant—boosts CD4^+^ T cell and T_fh_ cell activity to induce robust germinal centre responses (488). This leads to long-lived plasma and memory B cells, as well B cell repertoire expansion, resulting in elevated antibody titres. Importantly, however, MF59 may also support class switching to IgG in a CD4^+^ T cell-independent manner (489). Consequently, MF59 is a useful adjuvant for boosting responses in individuals experiencing reduced vaccine immunogenicity and has been successfully trialled in influenza vaccines for the elderly (490, 491), with especially pronounced benefits for elderly individuals with chronic comorbidities (492). Importantly, MF59 also induces a highly functional antibody response with selective induction of IgG3, resulting in pronounced boosting of Fc effector functions (394). An H5N1 avian influenza human vaccine immunogenicity trial revealed that participants adjuvanted with MF59 had elevated IgG1 and IgG3 titres, as well as ADNP and ADCD, in comparison to those boosted with alum (394). As such, MF59 may override the unfavourable subclass biases or impaired class switching experienced by certain vulnerable populations.

Non-human primate studies of MF59 compared to alum adjuvanted HIV-1 vaccines further point to the ability of adjuvant selection to regulate IgG glycosylation and Fc effector functions (493). A comparison of SIV vaccination with gp120, adjuvanted with either MF59 or alum indicated that although MF59 induced the expected higher titre antibody responses, the alum adjuvanted vaccine was associated with increased protection (493). The alum adjuvant induced decreased galactosylated IgG which was suggested to drive a more coordinated polyfunctional antibody response compared to MF59. On the other hand, MF59 induced elevated titres of anti-inflammatory sialylated gp120 antibodies. Indeed, observations in human trials of H5N1 vaccines suggested MF59 associated improvements to ADCP were driven by titre rather than Fc glycosylation modulation (394). In addition, the lack of enhanced FcγRIIIa engagement and ADCC despite increased IgG1 and IgG3, suggest an inability of MF59 to induce coordinated Fc functional responses, possibly associated with inhibitory Fc glycosylation (394).

In a second rhesus macaque study of a HIV-1 poxvirus vector vaccine administered with either alum alone or a liposomal monophosphoryl lipid A formulation plus alum (Army Liposome Formulation (ALFA)), the ALFA adjuvanted regimen was associated with enhanced ADNP and ADCP and 90% protection against mucosal challenge compared to the 100% infection risk observed for the alum adjuvanted regimen (494). The discrepancy between these studies regarding the effects of alum upon Fc functions may be related to use of different combinations of antigen choice, vaccine platform, or dosage. As such, these data underscore the need for detailed understanding of the interactions between various vaccine modifications upon polyfunctional antibodies.

Fortunately, a more detailed mechanistic understanding regarding how adjuvants alter IgG subclass ratios and IgG glycosylation is beginning to develop (288). Experimental water-in-oil emulsion and *Mycobacterium tuberculosis*-derived adjuvants appear to selectively program germinal centres to produce differentially Fc-glycosylated antibodies. Mouse model studies of the mechanism by which these adjuvants influence Fc functional antibodies identified unique transcriptome alterations distinguished by *St6gal1* mRNA levels which control sialyltransferase expression and, therefore, IgG Fc sialylation (288). The use of water-in-oil emulsion adjuvants and mycobacterium cord factor created germinal centre environments enriched in IL-6 which programmed T_fh_ cells to stimulate germinal centre B cells into producing IgG with reduced IgG Fc sialylation (288). Given the inflammatory properties of reduced Fc sialylation, inclusion of these adjuvants may support rational vaccine design targeting antibodies with enhanced effector functions. In addition, priming viral immunisation schedules with the unrelated TB Bacillus Calmette–Guérin (BCG) vaccine modulates production of IL-6, as well as other pro-inflammatory cytokines, upon target antigen stimulation (495–498). This suggests a possible role for BCG vaccination in the modulation of IgG Fc glycosylation, and consequently, regulation of Fc effector functions.

### Route of administration

Comparison of systemic versus mucosal vaccination is important for the many pathogens entering via the mucosa, such as HIV-1 and respiratory viruses. For example, for respiratory viruses, vaccines administered at the anatomical site of entry (i.e., intranasally) may elicit more biologically relevant, and therefore, protective, responses. However, traditionally, most vaccines are delivered intramuscularly, and historically have not facilitated optimal humoral immune responses at mucosal surfaces for influenza, SARS-CoV-2, and HIV-1 vaccination (499). Insights into the regulation of Fc effector functions can be gleaned from the comparison of intramuscular and aerosol delivery of a SIV vaccine in nonhuman primates (500). These two delivery modes of an otherwise identical vaccine mediated equivalent protection, but distinct effector functions. Intramuscular delivery facilitated enhanced IgG-driven ADCP, while aerosol delivery facilitated enhanced ADNP bolstered by IgA activity. Although a mechanism for this observation was not fully explored, shared patterns of IgG galactosylation were associated with the different modes of phagocytosis induced by each immunisation route, highlighting key glycoforms of potential clinical relevance (500).

In addition, combining different routes of administration within prime-boost vaccination regimens against infections where both mucosal and systemic protection is required may prove beneficial. Systems serology studies have shown COVID-19 convalescent vaccinated individuals induce markedly altered Fc functions compared to those exposed only to vaccination (501). Individuals with hybrid immunity (SARS-CoV-2 infected individuals who then received a single SARS-CoV-2 intramuscular vaccination) had increased Fc functional capacity in comparison to otherwise healthy individuals given two doses of intramuscular vaccination despite antibody titres being comparable between the two groups (501). This suggests combined aerosol and intramuscular vaccine delivery may be beneficial for the generation of enhanced functional antibody responses, potentially leading to improved protection. Alternatively, FcRn-targeting vaccines with the capacity to generate antibodies that are selectively transferred from systemic circulation to mucosal sites may overcome the issues associated with conflicting anatomical sites of vaccination and infection.

## Computational strategies to inform population-based vaccine design

Computational approaches can serve as safe, rapid, and cost-effective hypothesis testing tools to screen through multiple complex antibody scenarios, integrating and assessing for the influence of different geographic, genetic and clinical parameters that are rarely accounted for in traditional vaccine efficacy trials. The primary strength of these approaches is that they are able to integrate large amounts of complex data to gain insight into mechanisms that underpin variability in vaccine-induced protection (34, 502). Computational methods in systems serology can be divided into two groups, depending on the questions being asked and the data that is available.

Data-driven modelling involves the application of statistical and machine learning methods to high-throughput serology data to uncover ‘signatures’ of antibody features associated with a vaccine outcome (21). These methods can also be used to classify subpopulations of vaccinees based on responses within a given cohort (21). The advantage of data-driven approaches is that they require little prior knowledge of mechanism, making them broadly applicable to any data set of interest (21, 503). Data-driven approaches applied to plasma samples from the RV144, VAX003, HVTN204, and IPCAVD001 HIV vaccine trials identified antibody signatures that defined each vaccine response uniquely (34). Further, these approaches could also select for the humoral features (i.e., IgG titres, IgG-FcγR engagement) and functional responses (i.e., ADCP) most closely associated with protection against HIV infection (34). Results highlighted the key antibody features and functions that may be unique to each vaccine platform that was evaluated. Similarly, computational approaches applied to convalescent plasma data from COVID-19 patients revealed a signature of antibody features, primarily driven by SARS-CoV-2 Spike-specific IgG3 titres, that was associated with disease severity (504). Separate analysis of SARS-CoV-2 convalescent plasma samples has noted key differences in humoral profiles between children and elderly patients, with mature IgG and IgA responses to SARS-CoV-2 Spike 2 and Nuclear Protein antigens being associated more with elderly patients (37). These analysis reveals both the differences in immune signatures between populations and a possible reason for the vastly different clinical outcomes between them. In all, these findings illustrate how data-driven computational approaches can classify responses and identify subpopulations based on a distinct humoral signature rather than any single antibody feature.

In contrast, mechanistic (“theory-driven”) modelling requires knowledge of the underlying system and uses mathematical relationships to link system components. This trade-off sacrifices broad applicability for added depth of analysis. As these models describe the underlying system in detail, they allow for the investigation of the relationships between the components (e.g., antibodies and FcγR engagement) even at the individual level, thus allowing for the incorporation of personalised parameters (e.g., clinical history or immunogenetics) to evaluate how one individual may respond to a vaccine differently from another individual with different personalised parameters (446). Further, it can be used to evaluate not just how individual changes affect the system output (e.g., vaccine-induced antibody responses), but how combinations of changes to multiple system parameters (e.g., combinations of different immunogenetics and/or clinical history) can result in synergistic changes that are greater than the sum of individual perturbations.

Understanding these mechanistic details will be of high value for future efforts to optimise precision vaccines. For example, IgG1 allotypes and FcγR polymorphisms have the potential to influence protective responses *via* associated changes in antibody concentration and binding to Fc receptors as previously discussed in the sections above (60, 254). Mechanistic-modelling approaches have been applied to unravel mechanisms by which IgG1 allotypes and Fc receptor polymorphisms influence protective Fc effector functions following HIV-1 vaccination (445). An ordinary differential equation model illustrated how individuals with the G1m-1,3 IgG1 allotype would be predicted to be more responsive to changes in IgG1 concentration (titres) that arise from traditional boosting regimens, whereas G1m1 and G1m1,3 individuals may require a modification to IgG1-FcγR affinity (via glycosylation) to improve Fc effector functions. Furthermore, results suggested that Fc receptor engagement may be unaffected by FcγR polymorphisms until IgG titres reached a very high level, such as those that would be acquired with vaccine boosting. The model was also able to test vaccine design hypotheses in simulated populations of individuals with heterogeneous genetic compositions and suggest specific interventions that would be most effective. Combined, these insights provide specific target design criteria for vaccines tailored to different populations.

Improvements to these computational methods combined with broader application of the techniques will continue to increase the utility of computational approaches for population-based vaccine design. These approaches may directly inform current strategies, such as vaccine boosting, by identifying which populations may benefit most from a given intervention based on infection history, host genetics, or other clinical parameters that influence antibody levels. For vaccine parameters that are not yet modifiable, computational approaches will help prioritize targets for future modifications, overcoming challenges related to time and cost.

## Potential pitfalls of Fc functional antibody targeting vaccines

Eliciting potent Fc-functional responses via vaccination in immunologically vulnerable populations has clear benefits for the generation of durable, cross-reactive humoral protection. However, the potential to induce adverse antibody responses should also be acknowledged. Antibody-dependent enhancement (ADE) of disease has been observed following administration of the formalin inactivated RSV (505) and measles vaccines (506) as well as tetravalent dengue vaccine (507, 508). Although these vaccines did not specifically aim to elicit Fc functions, aberrant FcγR engagement appears to have facilitated viral replication within FcγR-expressing cells, and consequently, more severe disease outcomes when vaccinees encountered the virus (61). In the case of vaccines specifically designed to elicit potent Fc functions, careful delineation of protective versus pathogenic Fc responses is required to ensure vaccine safety (509).

Induction of beneficial Fc functional responses is a careful balancing act between protective and pathogenic inflammation. Downstream, Fc functions may trigger inflammatory cytokine release which in turn regulates further recruitment and programming of innate and adaptive immune cells (510). Notably, ADCC must balance viral clearance with immune activation and, therefore, if poorly regulated, can lead to increased morbidity and mortality during some diseases such as dengue fever (507, 508). Although ADCC may contribute to SARS-CoV-2 control (511, 512), uncoordinated Fc functions are a feature of COVID-19, contributing to excess FcγR mediated activation of the innate immune system and consequent induction of cytokine storms (513, 514). The requirement of precise induction of select Fc effector functions has also been demonstrated in protection against *Salmonella* Typhi (174). Following vaccination, ADNP and antibody-dependent neutrophil oxidative burst (ADNOB) were associated with vaccine-induced protection, whereas breakthrough infection was associated with elevated ADCD, ADCP, and ADNKA (174).

In addition, given the importance of Fc functions in protection against infectious diseases, several pathogens have evolved Fc evasion mechanisms (515–517). Notably, the decoy Fc receptors expressed by members of the herpes virus family, such as glycoproteins gp34 and gp68 in human cytomegalovirus (HCMV) and gE and gI in HSV (518–520), may present unique challenges for the development of vaccines aimed at eliciting protective Fc functional antibodies. Strategies to promote preferential engagement with host FcγRs rather than viral decoy receptors will need to be devised. Several HSV-1 and HSV-2 vaccine candidates have been trialled, but without sufficient efficacy for licensure (125, 521). Interestingly, individuals bearing different IgG1 allotypes have been shown to differently engage HSV decoy Fc receptors (435), suggesting possible differences in susceptibility to HSV infection. Efficacy of HCMV candidate vaccines has similarly been low, particularly in vulnerable target populations such as pregnant women and transplant recipients (522). However, it is suggested that generation of robust Fc effector functions may provide protective responses against infection. As such, it is important to consider that HCMV expresses similar decoy Fc receptors with preferential binding by some human IgG1 allotypes (523, 524) which may add further complexity to the design of vaccines targeting robust FcγR engagement.

## Conclusion and future perspectives

The success of current population-based vaccination strategies which prioritise immunocompromised and vulnerable individuals for early, additional, and/or high-dose vaccines has demonstrated the value of selectively tailoring immunisation programmes (15, 390, 391). Furthermore, this precedent lays the groundwork for what can be achieved, if more subtle immunological differences between distinct populations are considered in vaccine implementation and design.

**Figure 7:**
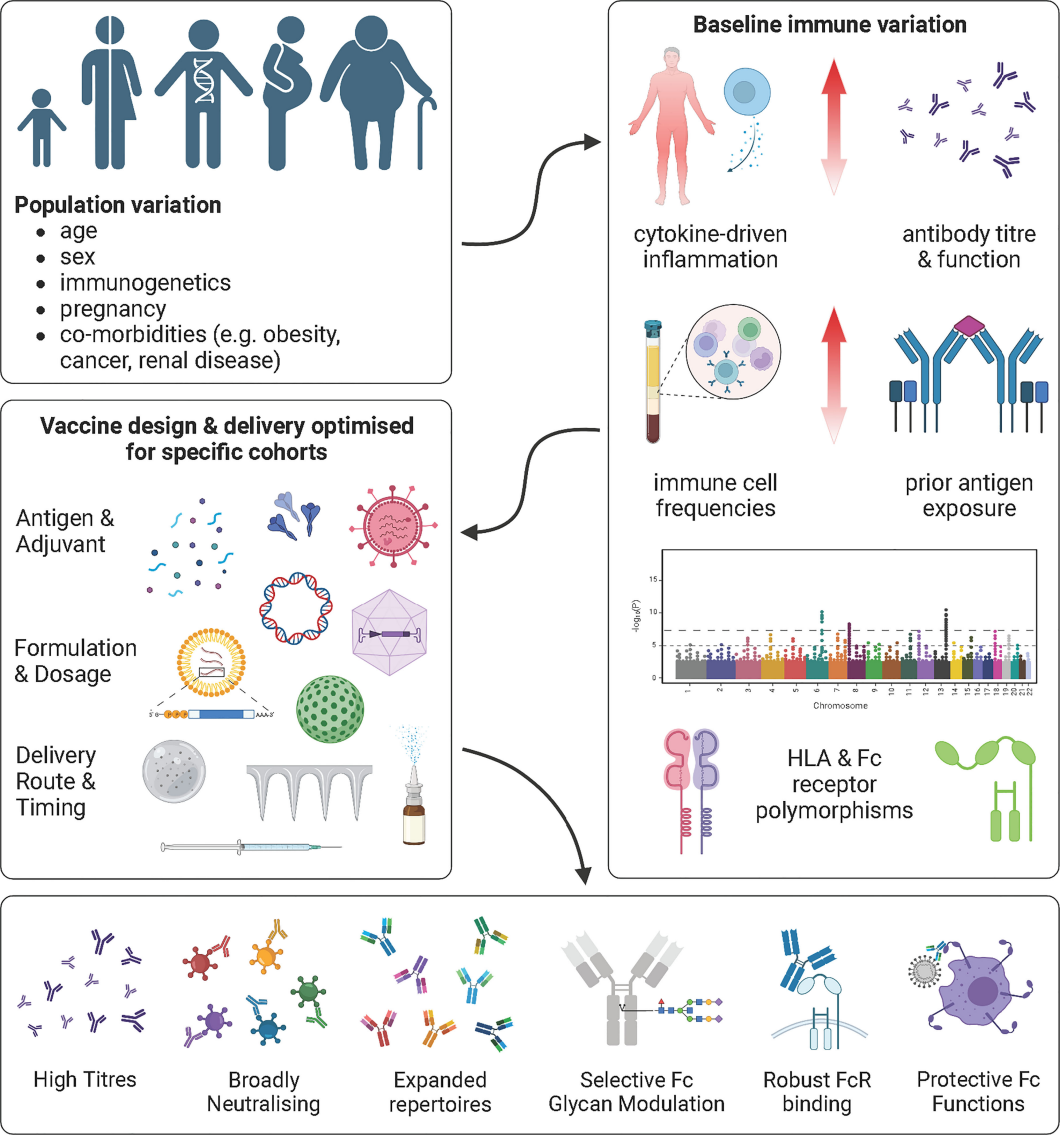
Considerations for the design of precision vaccines for vulnerable populations. Age, sex, immunogenetic, and baseline health variations within a vaccinated population can impact vaccine effectiveness. This population variation influences immune features known to modulate vaccine immunogenicity. However, precision vaccines designed to selectively boost the immune features that are impaired or dysregulated in vulnerable populations may enhance vaccine-induced protection. Design of such population-based precision vaccine strategies will require elucidation of the best combinations of antigen and adjuvant, vaccine formulation, and delivery mode in order to elicit an optimised polyfunctional antibody response and promote increased protection response.

Immunogenetic regulation, age- and disease-induced differences in host inflammatory status have emerged as potent modulators of vaccine-induced antibody responses (1) (**Figure 7**). Importantly, given that antibody features are similarly dysregulated across multiple vulnerable groups, there may be potential for broad implementation of population-based vaccination strategies aimed at bolstering protective Fc functions. For example, the elderly and individuals with chronic inflammatory comorbidities share similar dysregulated inflammatory Fc glycosylation states and impaired Fab affinity maturation (328, 330, 341). Therefore, future studies should consider focusing on the identification of appropriate adjuvants or vaccine platforms that modulate antibody glycosylation (288). In addition, strategies that increase antigen-specific antibody binding to FcγRIIb may be beneficial, given that FcγRIIb binding has been associated with enhanced neutralisation breadth as a result of increased affinity maturation (479). However, eliciting an optimally functional antibody response will require systematic assessment of the ideal combination of vaccine platform, adjuvant, dosage, and administrative route (**Figure 7**). Unfortunately, few clinical trials, or even licenced vaccine platforms, have assessed vulnerable groups for such nuanced variation. This highlights the need for more extensive population-based vaccine immunogenicity studies as well as standardisation of assays to assess functional antibody responses.

It is also important to note that the precise antibody features and effector functions constituting a coordinated Fc response are highly pathogen-specific. For example, while afucosylated IgG and excessive ADCC are detrimental for dengue fever (110, 111, 290, 292, 296), they are beneficial for HIV-1 and Tuberculosis (53, 79). Therefore, elucidation of the antibody features that should be targeted via vaccination will require detailed characterisation of the protective mechanisms employed against each disease, before being tailored to specific vulnerable populations. Despite the undeniable complexity of eliciting protective, polyfunctional antibodies, advances in vaccine formulation and administration may enable more precise modulation of IgG subclass ratios and Fc glycosylation which mitigate dysregulated Fc functions in vulnerable populations. However other target strategies should not be overlooked, such as the optimisation of FcRn engagement for vaccines which can be administered during pregnancy to simultaneously protect mother and infant, as well as systemic vaccines against mucosal pathogens.

Although personalised vaccination against infectious disease is likely not imminently practical at the individual level, population-based precision vaccination approaches appear feasible within the current global health infrastructure (5, 22, 525). Ideally, next-generation vaccination strategies will promote maximal responses not only in at-risk populations, but also in healthy individuals bearing genetic variations that necessitate differential boosting of specific immune features. Such population-based vaccination accounting for immunogenetic variation may be enabled by the ethnic and, therefore, geographic clustering of key heritable genetic features (313, 445, 446).

Finally, translating the observed differences between vaccine regimens into actionable vaccine design improvements for vulnerable populations remains a key public health priority. Indeed, a generation of population-based vaccination strategies informed by molecular mechanisms may enhance vaccine effectiveness against a broad range of diseases. Critically, such strategies may enable not only maximised protection of diverse, healthy individuals, but also markedly improved protection of the globally increasing population of immunologically vulnerable individuals.

## Author contributions

RP conceived and wrote the manuscript. RT contributed to writing. KA, AC, and KS edited the manuscript and supervised the project. All authors contributed to the article and approved the submitted version.
