# Supplementary material for: Polyfunctional antibodies: a path towards precision vaccines for vulnerable populations
Source: Front Immunol. 2023 Jun 27;14:1183727. doi: 10.3389/fimmu.2023.1183727 (PMC10433199; doi:10.3389/fimmu.2023.1183727)
Supplement: Supplementary file 1 [file Table_1.docx]

***Supplementary Material***

**Precision vaccines for vulnerable populations: The promise of polyfunctional antibodies**

**Ruth A Purcell^1^, Robert M Theisen^2^, Kelly B Arnold^2^, Amy W Chung^1^*, Kevin John Selva^1^***

^1^Department of Microbiology and Immunology, Peter Doherty Institute for Infection and Immunity, University of Melbourne, Melbourne, Victoria, 3000, Australia.

^2^Department of Biomedical Engineering, University of Michigan, Ann Arbor, MI, USA

| **Pathogen (Infectious disease)** | **Immunogenetic modulation of antibody and Fc functional responses** *(comparator genotype)* | | | **Comment** |
| --- | --- | --- | --- | --- |
|  | **IgG polymorphisms** | **FcγR polymorphisms/CNV** | **Other** |  |
| **Dengue virus (Dengue fever)** |  | **FcγRIIa-131 H/H & H/R:** 🡩 incidence of clinical infection *(FcγRIIa-131 R/R)* (1)  **FcγRIIa-131 R/R:** 🡩 protection against severe disease *(FcγRIIa-131 H/H)* (1) |  |  |
| ***Haemophilus influenzae* serotype b (Hib)** | **G2m(23):** 🡩 post-vaccination IgG titre & 🡫 infection risk *(G2m..)* (2)  **G2m(23):** 🡫 invasive disease susceptibility *(G2m..)* (3) |  | **C2 Complement deficiency:** 🡩 infection risk (4)* | *C2 deficiency to may impair responses via lectin pathway, as well as ADCD via classical pathway |
| **Hepatitis C virus (Hepatitis B)** | **Gm1,5,13,17:** 🡩 envelope glycoprotein E1E2 IgG titre (5) & 🡩 likelihood of infection clearance *(Non Gm1,5,13,17)* (6, 7) |  |  |  |
| **Hepatitis B virus (Hepatitis B)** | **G1m17:** 🡫 risk of chronic infection *(G1m3)* (8) |  |  |  |
| **Herpes Simplex Viruses 1 & 2** | **G1m1,17:** 🡩 IgG1-viral decoy FcγR engagement (9)* | **FcγRIIIa-158V/V:** 🡩 ADCC & 🡩 protection in **IgG1 G1m-1,3** homozygotes (10) |  | *May impair ADCC in **G1m1,17** homozygotes |
| **Human cytomegalovirus** | **G1m17:** 🡩 anti-glycoprotein B IgG titre *(G1m3)* (11)  **G1m1,17:** 🡩 IgG1-viral decoy FcγR gp68 engagement *(G1m-1,3)* (12)*  **G1m-1,3:** 🡩 IgG1-viral decoy FcγR gp34 engagement *(G1m1,17)* (13)* |  |  | *Increased Fc function antagonism of gp34 (14) may underpin predisposition of **G1m-1,3** individuals to HCMV-associated malignancies (15) |
| **Human immunodeficiency virus 1 (Acquired immunodeficiency syndrome)** | **G1m1:** 🡩 IgG1/IgG2 subclass ratio post-vaccination *(G1m3)* (16)  **G3m5*:** 🡩 likelihood of control *(G3m21*)* (17) | **FcγRIIa-131R/R:** 🡩 disease progression *(FcγRIIa-131H/H & FcγRIIa-131H/R)* (18)  **FcγRIIIa-158V/V:** 🡩 infection rate among low-infection risk vaccinees *(FcγRIIIa-158F/F)* (19)  **FcγRIIIa-158V/V:** 🡩 infection risk & disease progression *(FcγRIIIa-158F/F)* (20) |  |  |
| **Influenza B virus (Influenza)** | **G3m5*:** 🡩 IgG3 against H1N1 and H3N2 antigens *(G3m21*)* (21) |  |  |  |
| **Respiratory Syncytial Virus (RSV)** |  | **FcγRIIa-131 H/H:** 🡩 disease severity *(FcγRIIa-131 R/R)* (22) | **IL8 promoter polymorphism, −251A**: 🡩 IL-8, 🡩 Neutrophil infiltration, 🡩 disease susceptibility (23, 24) |  |
| **SARS-CoV-2 (COVID-19)** |  | **FcγRIIIa-158V/V:** 🡩 **ADCC** & 🡩 prevalence in hospitalised patients *(FcγRIIIa-158F)* (25) |  |  |
| ***Mycobacterium tuberculosis* (Tuberculosis)** |  | 🡩 ***FCGRI* copy number:** 🡩 likelihood of active compared to latent disease (26)  🡫 ***FCGRIIC* & *FCGRIIIB* copy number:** 🡩 likelihood infection in HIV^+^ patients (27) |  |  |
| ***Neisseria meningitidis* (Meningococcal disease)** |  | **FcγRIIa-131R/R:** 🡫 **ADNP** & 🡩 disease risk & severity *(FcγRIIa-131H/H)* (28, 29, 30) |  |  |
| ***Plasmodium falciparum* & *Plasmodium vivax* (Malaria)** | **G3m24 & G3m6:** 🡩 *P. falciparum* infection risk (31)  **Gm1,17,5,13,14,6:** 🡩 *P. falciparum* incidence, 🡩 baseline total IgG, 🡩 IgG2 & IgG4 (32)  **Gm3,23,5,13,14:** 🡩 anti-*Plasmodium vivax* merozoite surface protein-1 & apical membrane antigen-1 IgG1 titres (33)  **Gm5,6,13,14; 1,17:** 🡫 *P. falciparum* risk symptomatic infection but 🡫 likelihood of uncomplicated malaria (34) | **FcγRIIIa-158V/V:** 🡩 protection of Saudi children (35)  **FcγRIIb-232 T/T:** 🡫 disease susceptibility (36)  **FcγRIIIa-158V/V:** 🡩 infection risk in high exposure region (31)  **FcγRIIb I232T:** 🡩 protection against severe malaria (36) |  | A highly complex relationship exists between IgG allotypes and malaria risk. This most likely reflects the exceptionally strong evolutionary pressure exerted by malaria upon human populations for approximately 10,000 years and the consequent diversity in genetic adaptations that have arisen among different populations (37). As such, this list of immunogenetic influences upon malaria susceptibility and disease outcome is non-exhaustive. |
| **Poliovirus (Poliomyelitis)** |  | **FcγRIIIa-158F/F:** 🡩 disease risk (38) |  |  |
| ***Salmonella enterica* serotype Typhi (Typhoid fever)** | **Gm3,5,23:** 🡩 survival *(Gm1,2,17,21)* (39) |  |  |  |
| ***Streptococcus pneumoniae* (pneumococcal disease)** | **G2m(23):** 🡩 post-vaccination IgG titre *(G2m..)* (2) | **FcγRIIa-131H/H:** 🡩 ADNP *(FcγRIIa-131R/R)* (40) | **C2 Complement deficiency:** 🡩 infection risk (4)* | *C2 deficiency to may impair responses via lectin pathway, as well as ADCD via classical pathway |

**References**

1. Mohsin SN, Mahmood S, Amar A, Ghafoor F, Raza SM, Saleem M. Association of FcγRIIa Polymorphism with Clinical Outcome of Dengue Infection: First Insight from Pakistan. Am J Trop Med Hyg. 2015;93(4):691-6.

2. Ambrosino DM, Schiffman G, Gotschlich EC, Schur PH, Rosenberg GA, DeLange GG, et al. Correlation between G2m(n) immunoglobulin allotype and human antibody response and susceptibility to polysaccharide encapsulated bacteria. J Clin Invest. 1985;75(6):1935-42.

3. Goddard EA, Beatty DW, Hoffman EB. Immunoglobulin allotypes and genetic susceptibility to invasive Haemophilus influenzae type b and Staphylococcus aureus infections in South African children. Pediatr Infect Dis J. 1996;15(5):419-24.

4. Fasano MB, Hamosh A, Winkelstein JA. Recurrent systemic bacterial infections in homozygous C2 deficiency. Pediatric Allergy and Immunology. 1990;1(1):46-9.

5. Pandey JP, Luo Y, Elston RC, Wu Y, Philp FH, Astemborski J, et al. Immunoglobulin allotypes influence IgG antibody responses to hepatitis C virus envelope proteins E1 and E2. Hum Immunol. 2008;69(3):158-64.

6. Pandey JP, Astemborski J, Thomas DL. Epistatic effects of immunoglobulin GM and KM allotypes on outcome of infection with hepatitis C virus. J Virol. 2004;78(9):4561-5.

7. Pandey JP, Namboodiri AM, Luo Y, Wu Y, Elston RC, Thomas DL, et al. Genetic markers of IgG influence the outcome of infection with hepatitis C virus. J Infect Dis. 2008;198(9):1334-6.

8. Di Bona D, Pandey JP, Aiello A, Bilancia M, Candore G, Caruso C, et al. The immunoglobulin γ marker 17 allotype and KIR/HLA genes prevent the development of chronic hepatitis B in humans. Immunology. 2020;159(2):178-82.

9. Atherton A, Armour KL, Bell S, Minson AC, Clark MR. The herpes simplex virus type 1 Fc receptor discriminates between IgG1 allotypes. Eur J Immunol. 2000;30(9):2540-7.

10. Moraru M, Black LE, Muntasell A, Portero F, López-Botet M, Reyburn HT, et al. NK Cell and Ig Interplay in Defense against Herpes Simplex Virus Type 1: Epistatic Interaction of CD16A and IgG1 Allotypes of Variable Affinities Modulates Antibody-Dependent Cellular Cytotoxicity and Susceptibility to Clinical Reactivation. J Immunol. 2015;195(4):1676-84.

11. Pandey JP, Kistner-Griffin E, Radwan FF, Kaur N, Namboodiri AM, Black L, et al. Immunoglobulin genes influence the magnitude of humoral immunity to cytomegalovirus glycoprotein B. J Infect Dis. 2014;210(11):1823-6.

12. Pandey JP, Namboodiri AM, Radwan FF, Nietert PJ. The decoy Fcγ receptor encoded by the cytomegalovirus UL119-UL118 gene has differential affinity to IgG proteins expressing different GM allotypes. Hum Immunol. 2015;76(8):591-4.

13. Namboodiri AM, Pandey JP. The human cytomegalovirus TRL11/IRL11-encoded FcγR binds differentially to allelic variants of immunoglobulin G1. Arch Virol. 2011;156(5):907-10.

14. Corrales-Aguilar E, Trilling M, Hunold K, Fiedler M, Le VT, Reinhard H, et al. Human cytomegalovirus Fcγ binding proteins gp34 and gp68 antagonize Fcγ receptors I, II and III. PLoS Pathog. 2014;10(5):e1004131.

15. Pandey JP. Immunoglobulin GM Genes, Cytomegalovirus Immunoevasion, and the Risk of Glioma, Neuroblastoma, and Breast Cancer. Front Oncol. 2014;4:236.

16. Kratochvil S, McKay PF, Chung AW, Kent SJ, Gilmour J, Shattock RJ. Immunoglobulin G1 Allotype Influences Antibody Subclass Distribution in Response to HIV gp140 Vaccination. Front Immunol. 2017;8:1883.

17. Deepe RN, Kistner-Griffin E, Martin JN, Deeks SG, Pandey JP. Epistatic interactions between Fc (GM) and FcγR genes and the host control of human immunodeficiency virus replication. Hum Immunol. 2012;73(3):263-6.

18. Forthal DN, Landucci G, Bream J, Jacobson LP, Phan TB, Montoya B. FcgammaRIIa genotype predicts progression of HIV infection. J Immunol. 2007;179(11):7916-23.

19. Forthal DN, Gabriel EE, Wang A, Landucci G, Phan TB. Association of Fcγ receptor IIIa genotype with the rate of HIV infection after gp120 vaccination. Blood. 2012;120(14):2836-42.

20. Poonia B, Kijak GH, Pauza CD. High affinity allele for the gene of FCGR3A is risk factor for HIV infection and progression. PLoS One. 2010;5(12):e15562.

21. Hensen L, Nguyen THO, Rowntree LC, Damelang T, Koutsakos M, Aban M, et al. Robust and prototypical immune responses toward influenza vaccines in the high-risk group of Indigenous Australians. Proc Natl Acad Sci U S A. 2021;118(41).

22. Holgado MP, Raiden S, Sananez I, Seery V, De Lillo L, Maldonado LL, et al. Fcγ Receptor IIa (FCGR2A) Polymorphism Is Associated With Severe Respiratory Syncytial Virus Disease in Argentinian Infants. Front Cell Infect Microbiol. 2020;10:607348.

23. Lu A, Wang L, Zhang X. Haplotype of IL-8 -251T and 781C is associated with the susceptibility to respiratory syncytial virus. J Trop Pediatr. 2010;56(4):242-6.

24. Hull J, Thomson A, Kwiatkowski D. Association of respiratory syncytial virus bronchiolitis with the interleukin 8 gene region in UK families. Thorax. 2000;55(12):1023-7.

25. Vietzen H, Danklmaier V, Zoufaly A, Puchhammer-Stöckl E. High-affinity FcγRIIIa genetic variants and potent NK cell-mediated antibody-dependent cellular cytotoxicity (ADCC) responses contributing to severe COVID-19. Genet Med. 2022;24(7):1449-58.

26. Sutherland JS, Loxton AG, Haks MC, Kassa D, Ambrose L, Lee JS, et al. Differential gene expression of activating Fcγ receptor classifies active tuberculosis regardless of human immunodeficiency virus status or ethnicity. Clin Microbiol Infect. 2014;20(4):O230-8.

27. Machado LR, Bowdrey J, Ngaimisi E, Habtewold A, Minzi O, Makonnen E, et al. Copy number variation of Fc gamma receptor genes in HIV-infected and HIV-tuberculosis co-infected individuals in sub-Saharan Africa. PLoS One. 2013;8(11):e78165.

28. Sanders LA, Feldman RG, Voorhorst-Ogink MM, de Haas M, Rijkers GT, Capel PJ, et al. Human immunoglobulin G (IgG) Fc receptor IIA (CD32) polymorphism and IgG2-mediated bacterial phagocytosis by neutrophils. Infect Immun. 1995;63(1):73-81.

29. Fijen CA, Bredius RG, Kuijper EJ, Out TA, De Haas M, De Wit AP, et al. The role of Fcgamma receptor polymorphisms and C3 in the immune defence against Neisseria meningitidis in complement-deficient individuals. Clin Exp Immunol. 2000;120(2):338-45.

30. Platonov AE, Shipulin GA, Vershinina IV, Dankert J, van de Winkel JG, Kuijper EJ. Association of human Fc gamma RIIa (CD32) polymorphism with susceptibility to and severity of meningococcal disease. Clin Infect Dis. 1998;27(4):746-50.

31. Fall A, Dechavanne C, Sabbagh A, Guitard E, Milet J, Garcia A, et al. Susceptibility to Plasmodium falciparum Malaria: Influence of Combined Polymorphisms of IgG3 Gm Allotypes and Fc Gamma Receptors IIA, IIIA, and IIIB. Front Immunol. 2020;11:608016.

32. Giha HA, Nasr A, Iriemenam NC, Arnot D, Troye-Blomberg M, Theander TG, et al. Antigen-specific influence of GM/KM allotypes on IgG isotypes and association of GM allotypes with susceptibility to Plasmodium falciparum malaria. Malar J. 2009;8:306.

33. Pandey JP, Morais CG, Fontes CJ, Braga EM. Immunoglobulin GM 3 23 5,13,14 phenotype is strongly associated with IgG1 antibody responses to Plasmodium vivax vaccine candidate antigens PvMSP1-19 and PvAMA-1. Malar J. 2010;9:229.

34. Migot-Nabias F, Noukpo JM, Guitard E, Doritchamou J, Garcia A, Dugoujon JM. Imbalanced distribution of GM immunoglobulin allotypes according to the clinical presentation of Plasmodium falciparum malaria in Beninese children. J Infect Dis. 2008;198(12):1892-5.

35. Nasr A, Aljada A, Hamid O, Elsheikh HA, Masuadi E, Al-Bawab A, et al. Significant differences in FcγRIIa, FcγRIIIa and FcγRIIIb genes polymorphism and anti-malarial IgG subclass pattern are associated with severe Plasmodium falciparum malaria in Saudi children. Malar J. 2021;20(1):376.

36. Willcocks LC, Carr EJ, Niederer HA, Rayner TF, Williams TN, Yang W, et al. A defunctioning polymorphism in FCGR2B is associated with protection against malaria but susceptibility to systemic lupus erythematosus. Proc Natl Acad Sci U S A. 2010;107(17):7881-5.

37. Kwiatkowski DP. How malaria has affected the human genome and what human genetics can teach us about malaria. Am J Hum Genet. 2005;77(2):171-92.

38. Rekand T, Langeland N, Aarli JA, Vedeler CA. Fcgamma receptor IIIA polymorphism as a risk factor for acute poliomyelitis. J Infect Dis. 2002;186(12):1840-3.

39. de Vries RR, Meera Khan P, Bernini LF, van Loghem E, van Rood JJ. Genetic control of survival in epidemics. J Immunogenet. 1979;6(4):271-87.

40. Rodriguez ME, van der Pol W-L, Sanders LAM, van de Winkel JGJ. Crucial Role of FcγRIIa (CD32) in Assessment of Functional Anti—Streptococcus pneumoniae Antibody Activity in Human Sera. The Journal of Infectious Diseases. 1999;179(2):423-33.
